# Temporal changes in mouse hippocampus transcriptome after pilocarpine-induced seizures

**DOI:** 10.3389/fnins.2024.1384805

**Published:** 2024-07-08

**Authors:** Evgenya Y. Popova, Yuka Imamura Kawasawa, Ming Leung, Colin J. Barnstable

**Affiliations:** ^1^Department of Neural and Behavioral Sciences, Penn State University College of Medicine, Hershey, PA, United States; ^2^Penn State Hershey Eye Center, Hershey, PA, United States; ^3^Department of Pharmacology, Penn State University College of Medicine, Hershey, PA, United States; ^4^Center for Cancer Genomics and Precision Oncology, Wake Forest Baptist Comprehensive Cancer Center, Winston Salem, NC, United States

**Keywords:** hippocampus, status epilepticus, pilocarpine, mRNA-seq, gene expression, microRNA

## Abstract

**Introduction:**

Status epilepticus (SE) is a seizure lasting more than 5 min that can have lethal consequences or lead to various neurological disorders, including epilepsy. Using a pilocarpine-induced SE model in mice we investigated temporal changes in the hippocampal transcriptome.

**Methods:**

We performed mRNA-seq and microRNA-seq analyses at various times after drug treatment.

**Results:**

At 1 h after the start of seizures, hippocampal cells upregulated transcription of immediate early genes and genes involved in the IGF-1, ERK/MAPK and RNA-PolII/transcription pathways. At 8 h, we observed changes in the expression of genes associated with oxidative stress, overall transcription downregulation, particularly for genes related to mitochondrial structure and function, initiation of a stress response through regulation of ribosome and translation/EIF2 signaling, and upregulation of an inflammatory response. During the middle of the latent period, 36 h, we identified upregulation of membrane components, cholesterol synthesis enzymes, channels, and extracellular matrix (ECM), as well as an increased inflammatory response. At the end of the latent period, 120 h, most changes in expression were in genes involved in ion transport, membrane channels, and synapses. Notably, we also elucidated the involvement of novel pathways, such as cholesterol biosynthesis pathways, iron/BMP/ferroptosis pathways, and circadian rhythms signaling in SE and epileptogenesis.

**Discussion:**

These temporal changes in metabolic reactions indicate an immediate response to injury followed by recovery and regeneration. CREB was identified as the main upstream regulator. Overall, our data provide new insights into molecular functions and cellular processes involved at different stages of seizures and offer potential avenues for effective therapeutic strategies.

## Introduction

It is estimated that 1 in 10 people will experience a seizure at some point in their lives. In the US alone, there are 3.4 million people with chronic epilepsy, including 400,000 children (Zack and Kobau, 2017). In 2022 the World Health Organization estimated the number of people with epilepsy globally exceeds 50 million.

Despite its prevalence and substantial research efforts, the etiology and pathogenesis of seizures remain poorly understood and around 30% of seizures are drug resistant (Walker, 2018). In the past decades, the use of rodent models of epilepsy has allowed the observation of morphological differences in brain structures, characterization of behavioral phenotypes, and quantification of electrophysiological changes. Early studies have implicated the hippocampus is involved in both the generation of seizure activity and the location of post-seizure injuries for various types of seizures, including temporal lobe epilepsy (TLE) (Schwartzkroin, 1994).

Among the various rodent models of epilepsy, a mouse model of TLE induced by the chemoconvulsant pilocarpine has been widely used due to its close resemblance to human TLE. It is robust while cost-effective, making it suitable for testing the efficacy of novel therapies for severe TLE in rodents, where existing anticonvulsant is not effective in humans. Pilocarpine, when administered subcutaneously or intraperitoneally in rodents, is known to induce a condition of prolonged seizures called Status Epilepticus (SE), which is considered an acute characteristic of this model. In human patients, SE is defined as one or more seizures within a period of 5 min, with no return to normal consciousness between events. SE is a life-threatening condition that can result in permanent brain damage or death, and it can arise not only from pre-existing poorly controlled epilepsy but also from other brain problems such as trauma, stroke, or infection. A history of having an SE has been linked to future development of TLE, and therefore, characterization of molecular changes in the hippocampus after the systematic induction of SE in rodent models can provide important insights to the etiology of epileptogenesis and potential targets of novel interventions to pharmacoresistant TLE.

More recently, advances in technology have sparked interest in characterizing gene expression related to seizures and epileptogenesis. Genetic methods such as quantitative real time PCR (qRT-PCR), microarrays, and mRNA-seq allow for tracking of gene expression at specific time points following seizures (Conte et al., 2020). When combined with manipulation of animal models, these methods offer the opportunity to extensively characterize alterations in gene expression that may be associated with epileptogenesis following seizure events (Dingledine et al., 2017). A number of studies have observed that gene modulation in the hippocampus continues for many days after the initial seizure event (Clasadonte et al., 2016). To determine earlier critical changes in the hippocampal gene expression, we utilized the pilocarpine-induced seizure model in C57BL/6 J mice and analyzed multiple time points over a period of five days. In addition to mRNA changes, we also observed a sequence of miRNA changes that may play a role in regulating gene expression.

## Methods

### Reagents

Bacteriostatic 0.9% sodium chloride was from APP Pharmaceuticals (Schaumburg, IL), pilocarpine hydrochloride was from MilliporeSigma (P04272-10 g; Burlington, MA), scopolamine hydrochloride was from Santa Cruz Biotechnology (sc-253544, Dallas, TX), 10x PBS was from Mediatech (46-013-CM, Corning, NY).

### Animals

Wild type C57BL/6 J (cat # 000664) mice were purchased from Jackson laboratory (Bar Harbor, ME) and housed in a room maintained at an ambient temperature of 25°C, with a humidity range of 30–70%. The mice were kept on a 12-h light–dark cycle and had *ad libitum* access to rodent chow. All animal experiments in this study were carried out in accordance with the National Research Council’s Guide for the Care and Use of Laboratory Animals (8th edition), and were approved by the Pennsylvania State University College of Medicine Institutional Animal Care and Use Committee (protocol #46432).

### Pilocarpine-mediated model of epileptic seizures

We have optimized the protocol of the pilocarpine-mediated model of epileptic seizures, with a focus on the neurodegeneration process in the hippocampus according to current literature. Male and female mice, aged 6–7 weeks, with a C57BL/6 J background and weighing 21–25 g, were first injected intraperitoneal (ip) with 1 mg/kg scopolamine to reduce peripheral cholinergic effects, followed 30 min later by first ip pilocarpine (350 mg/kg) or vehicle (0.9% bacteriostatic sodium chloride) administration (Borges et al., 2003; Buckmaster and Haney, 2012). Animals were closely monitored for seizure activity by direct observation of SE occurrence, stage of SE and mice health, and ip pilocarpine was administered in a ramping-up manner with 25-50 mg/kg does every 30 min until achieving seizures of at least stage 3, lasting for at least 45 min without full recovery in between (Schauwecker, 2012; Leung et al., 2015). Seizure stages were classified according to Borges et al. (2003): normal activity (stage 0); rigid posture (stage 1); stiffened, extended, and often arched tail (stage 2); partial body clonus or head bobbing (stage 3); whole body continuous clonic seizures while retaining posture (stage 3.5); rearing (stage 4); severe whole body continuous clonic seizures while retaining posture (stage 4.5); rearing and falling (stage 5); and tonic–clonic seizures with loss of posture or jumping (stage 6). Animals were closely monitored, kept warm, fed with moistened chow, and sacrificed at different time points after the induction of seizures (Supplementary Table S1). In our experiments SE usually continued for 60–90 min and we chose a time line to investigate early events in epileptogenesis before onset of seizures, namely immediately after seizure initiation (1 h), after the status epilepticus (SE) phase (8 h), during the middle of latent period (36 h), and at the end of latent period (120 h) before spontaneous and recurrent seizures occur. These time points closely resemble those used in other studies (Dingledine et al., 2017; Ahmed et al., 2021).

### Hippocampus isolation

Animals were humanely euthanized in their home cage with a controlled level of CO2 via a Euthenex CO2 flow regulator which monitors and controls gaseous flow rate to minimize animal discomfort and pain. Euthenex CO2 station has a regulator and flow meter installed in order to control the flow rate. The regulator is automatically set to match mouse cage. The regulator is set to provide a rate of 10–30% volume displacement per minute. No more than 2 animals were euthanized at a time. After the animal was sacrificed, it was decapitated and the brain was removed from the skull. Hippocampus isolation was carried out in PBS, following the protocol from Zurich University, Institute of Pharmacology and Toxicology “Dissection of adult mouse hippocampus from fresh tissue,” EJN protocols, 2014: https://www.youtube.com/watch?v=Upf15CB29V4. The right and left halves of the hippocampus were flash frozen in liquid nitrogen and stored in separate tubes at −80°C.

### mRNA-seq

We carried out mRNA-seq of hippocampus tissue isolated 1 h, 8 h, 36 h and 120 h after seizure induction in C57BL/6 J mice (Supplementary Table S1). Total RNA isolation was carried out from the left hippocampus with Trizol reagent (15,596,018, Thermo Fisher Scientific, Waltham, MA) as described in the vendor’s manual. The quality of RNA was assessed on an Agilent Bioanalyzer (Agilent Technologies, Santa Clara, CA) and confirmed that their RNA integrity numbers were greater than 7.9. The cDNA libraries were prepared using the Illumina Stranded mRNA Prep, Ligation kit (Illumina, San Diego, CA) as per the manufacturer’s instructions. Briefly, polyA-RNA was purified from 200 ng of total RNA using oligo (dT) beads. The extracted mRNA fraction was subjected to fragmentation, reverse transcription, end repair, 3′– end adenylation, and adaptor ligation, followed by PCR amplification and SPRI bead purification (Beckman Coulter, Brea, CA). The unique dual index sequences (IDT for Illumina RNA UD Indexes Set A, Ligation, Illumina) were incorporated in the adaptors for multiplexed high-throughput sequencing. The final product was assessed for its size distribution and concentration using BioAnalyzer High Sensitivity DNA Kit (Agilent Technologies). The libraries were pooled and diluted to 3 nM using 10 mM Tris–HCl, pH 8.5, and then denatured using the Illumina protocol. The denatured libraries were loaded onto an S1 flow cell on a NovaSeq 6,000 or a Rapid Flow cell on a HiSeq 2500 (Illumina) and run for 2×53 cycles according to the manufacturer’s instructions. De-multiplexed and adapter-trimmed sequencing reads were generated using Illumina bcl2fastq (released version 2.20.0) allowing no mismatches in the index read. BBDuk was used to trim/filter low-quality sequences using the “qtrim = lr trimq = 10 maq = 10” option. Next, alignment of the filtered reads to the mouse reference genome [mouse Ensembl release 67 (GRCm37/NCBIM37/mm9)] was done using HISAT2 (version 2.1.0) applying —no-mixed and —no-discordant options. Read counts were calculated using HTSeq by supplementing Ensembl gene annotation (release 67: “Mus_musculus.Ensembl.NCBIM37.67.gtf”). The edgeR R package was used to fit the read counts to the negative binomial model along with the generalized linear model (GLM) and differentially expressed genes were determined by the likelihood ratio test method implemented in the edgeR. Significance was defined to be those with *q*-value <0.05 calculated by the Benjamini-Hochberg method to control the false discovery rate (FDR). The ggplot2 R package was used for generating heatmaps. Raw fastq and counts data generated during this study are available at GEO (GSE198498). We then tested the relations and network interactions between gene functional groups using DAVID, KEGG, and Ingenuity Pathway Analysis (IPA, Qiagen, Germantown, MD). For functional enrichment analysis, we used more a relaxed significance cutoff: Ingenuity Pathway Analysis (IPA) with a *q*-value <0.5, and DAVID/GO analysis and KEGG analysis with a *p*-value <0.05. R package ClusterProfiler (Yu et al., 2012) was further used to summarize Gene Ontology and KEGG results. To examine how metabolic pathways changed with time, we used –Log (*p*-value) from IPA analysis as a measure of involvement of a particular metabolic pathway at different time points in response to seizure in the hippocampus. Additionally, we used CEMiTool (Co-Expression Modules identification Tool) to identify co-expression gene modules by setting *p*-value cutoff at 0.05 to filter most variant genes, followed by module correlation analysis by Pearson method (Russo et al., 2018).

### MicroRNA-seq

We carried out microRNA-seq (also known as small RNA-seq) of hippocampus tissue isolated 1 h, 8 h, 24 h and 120 h after seizure induction in C57BL/6 J mice (Supplementary Table S1). Total RNA ~2 μg was isolated from tissue using the Trizol method. Samples were analyzed using an Agilent Bioanalyzer 2,100 and only those with high integrity number (>7.5). Small RNA-sequencing libraries were prepared from 1–100 ng total RNA using the NEXTflex^™^ Small RNA Library Prep Kit v3 (Perkin Elmer, Waltham, MA) as per the manufacturer’s instructions. The unique barcode sequences were incorporated in the adaptors for multiplexed high-throughput sequencing. The final product was assessed for its size distribution and concentration using BioAnalyzer High Sensitivity DNA Kit (Agilent Technologies). The libraries were pooled and diluted to 3 nM using 10 mM Tris–HCl, pH 8.5 and then denatured using the Illumina protocol. The denatured libraries were loaded onto an S1 flow cell on a NovaSeq 6,000 or a Rapid flow cell on a HiSeq2500 (Illumina) and run for 1×49-65 cycles according to the manufacturer’s instructions. De-multiplexed sequencing reads were generated using Illumina bcl2fastq (released version 2.20.0.422, Illumina) allowing no mismatches in the index read. After applying the quality filtering, quality trimming and adapter trimming functions of FASTX-Toolkit’s,^1^ four bases were trimmed from both 5′ and 3′ ends of the sequencing reads using the fastq_trimmer function. Oasis 2.0 suite was used to align and count raw reads of all expressed small RNAs, followed by differential expression analysis and functional enrichment analysis of miRNAs using g:Profiler (GO, pathway-KEGG, Reactome) (Raudvere et al., 2019). To evaluate which genes or pathways might be regulated by microRNA from our data set we used miRTargetLink 2.0 (Kern et al., 2021). Raw counts and differential expression analysis generated during this study are available at GEO (GSE198498). All of the next-generation sequencing and data analysis were carried out using the Genome Sciences and Bioinformatics Core Facility at the Penn State College of Medicine (Facility RRID: SCR_021123).

### cDNA preparation

Final RNA concentrations were determined spectrophotometrically using a NanoDrop 1,000 Spectrophotometer (Thermo Fisher Scientific). cDNA was synthesized with SuperScript^™^ III First-Strand Synthesis System kit according to manufacturer’s protocol (Thermo Fisher Scientific).

### qRT-PCR

Primers were designed and purchased from Integrated DNA Technologies (IDT, Coralville, IA). The sequence information is listed in Supplementary Table S2. For quantitative real-time PCR, the 2x iQ^M^ SYBR® Green PCR supermix from Bio-Rad (Bio-Rad Laboratories, Hercules, CA) was used. Samples were run in triplicate on an iQ^M^5 Multicolor Real Time PCR Detection System (Bio-Rad Laboratories). The relative expression level of each gene was calculated by the 2^-∆∆Ct^ method and normalized to *Gapdh*. Genes were considered up-or down-regulated if *p* value was <0.05.

### Western blot

The right and left halves of the hippocampus were flash frozen in liquid nitrogen and stored in separate tubes at −80°C. Samples for SDS-PAGE were prepared by homogenizing the right halve in 500 μL of Lysis buffer (1% SDS, 10 mM EDTA, 50 mM Tris HCl pH8.0, 10 mM PMSF) and brief sonication, followed by the addition of Laemmli sample buffer. The samples were then boiled for 10 min, resolved on Criterion TGX Precast Gel AnyKD (Bio-Rad Laboratories), and transferred to a nitrocellulose membrane for immunoblotting. Antibodies against PTPN11 (SHP2, Cell Signaling Technology, Danvers, MA, D50F2) and FOS (Abcam, Cambridge, UK, 214672) were used at a dilution of 1:1000, while antibodies against ACTB were used at a dilution of 1:5000, and antibodies against GFAP (MilliporeSigma, MAB360) and pERK (Santa Cruz, sc-7383) were used at dilutions of 1:10000 and 1:500, respectively. Secondary goat antibodies against rabbit/mouse HRP (Jackson Immuno-Research Laboratory, West Grove, PA) were diluted as 1:5000. The ECL western blot detection system (Thermo Fisher Scientific) was used to visualize the binding of the primary antibodies. Autoradiographs after ECL detection were scanned and digitized and intensity of protein bands was quantitated using ImageJ software.

### Statistical analyses

Results are presented as means ± standard error of the mean (SEM). Unpaired, one tail Student’s t-test (two-tailed, unpaired) was used to evaluated statistical significance between groups. Experiments were conducted with two to four biological and three technical replicates. *p* value <0.05 was considered significant. Statistical analyses for experiments were performed using the GraphPad Prism software.

## Results

### mRNA-seq

**Figure 1:**
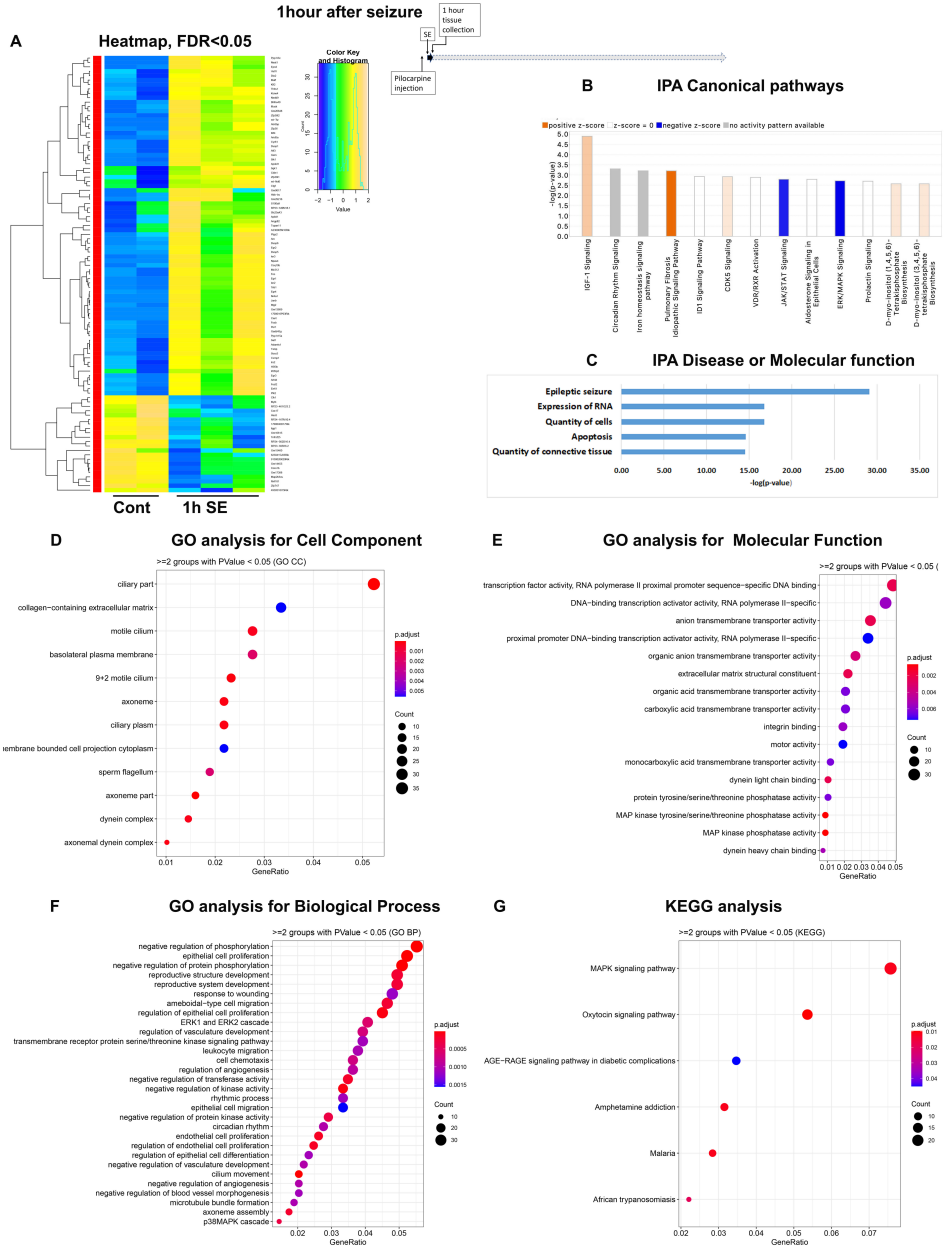
mRNA-seq analysis of changes in gene expression in mouse hippocampus 1 h after the induction of SE. **(A)** Heat map of differentially expressed genes (DEGs) between control and SE mice, with False Discovery Rate (FDR) < 0.05. **(B)** IPA Canonical Pathways that are significantly enriched for DEGs between control and SE mice, with z-score > 2.0 and FDR < 0.5. **(C)** IPA Molecular Function or Disease that are significantly enriched for DEG between control and SE mice (FDR < 0.5). **(D–F)** Gene Ontology (GO) functional enrichment analyses of DEGs between control and SE mice, categorized by Cell Component **(D)**, Molecular Function **(E)**, and Biological Process **(F)** with a significance threshold of *p* < 0.05. **(G)** KEGG pathway enrichment analysis of DEGs between control and SE mice with a significance threshold of *p* < 0.05. ClusterProfiler was used to identify significantly enriched GO or KEGG categories (*p* < 0.05), group them together based on semantic similarity, and visualize the results using a dotplot. The color scale represents the adjusted *p*-value, and the dot size represents the gene count in each term.

**Figure 2:**
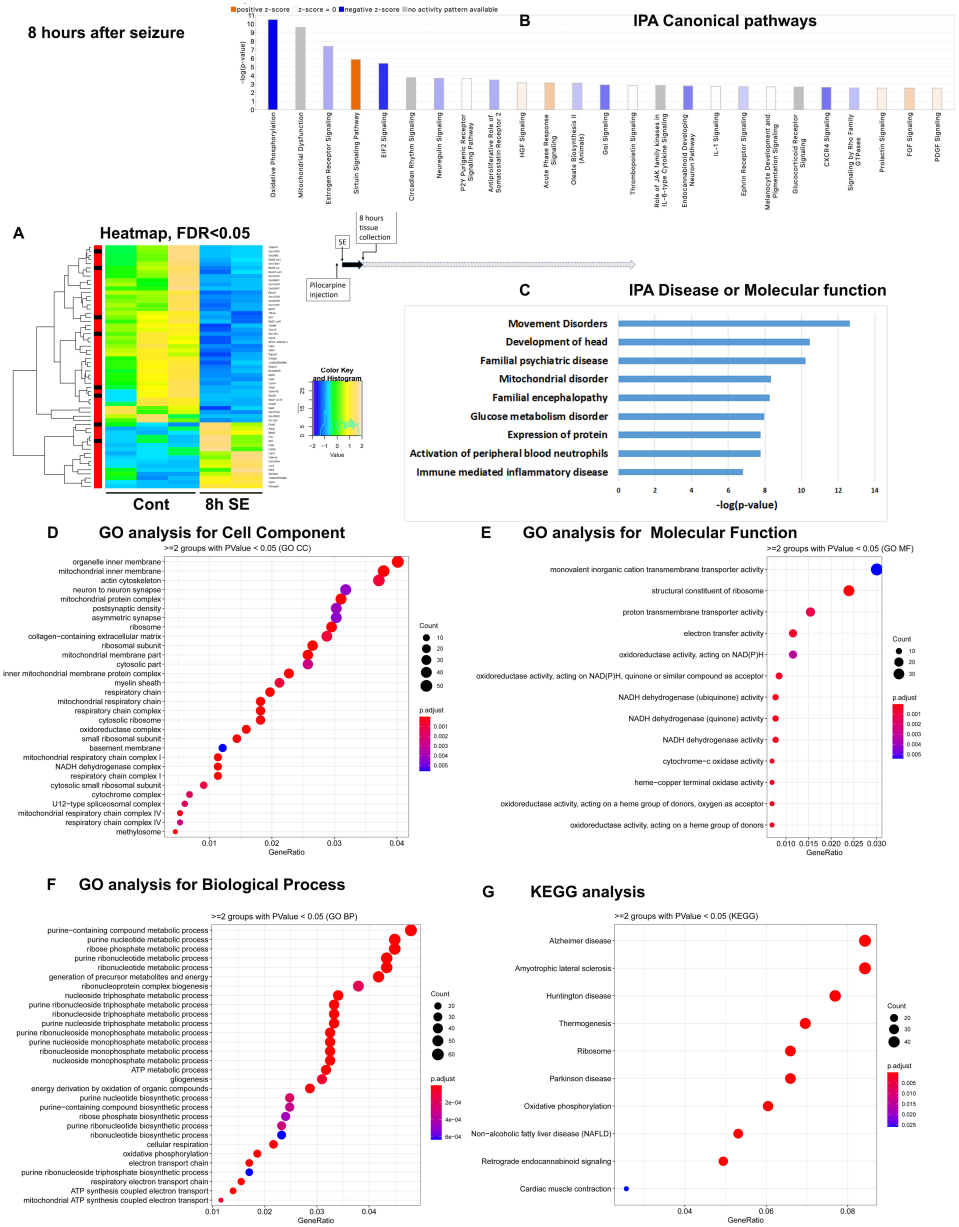
mRNA-seq analysis of changes in gene expression in mice hippocampus 8 h after the induction of SE. **(A)** Heat map of differentially expressed genes (DEGs) between control and SE mice, with False Discovery Rate (FDR) < 0.05. **(B)** IPA Canonical Pathways that are significantly enriched for DEGs between control and SE mice, with z-score > 2.0 and FDR < 0.5. **(C)** IPA Molecular Function or Disease that are significantly enriched for DEG between control and SE mice (FDR < 0.5). **(D–F)** Gene Ontology (GO) functional enrichment analyses of DEGs between control and SE mice, categorized by Cell Component **(D)**, Molecular Function **(E)**, and Biological Process **(F)** with a significance threshold of *p* < 0.05. **(G)** KEGG pathway enrichment analysis of DEGs between control and SE mice with a significance threshold of *p* < 0.05. ClusterProfiler was used to identify significantly enriched GO or KEGG categories (*p* < 0.05), group them together based on semantic similarity, and visualize the results using a dotplot. The color scale represents the adjusted *p*-value, and the dot size represents the gene count in each term.

**Figure 3:**
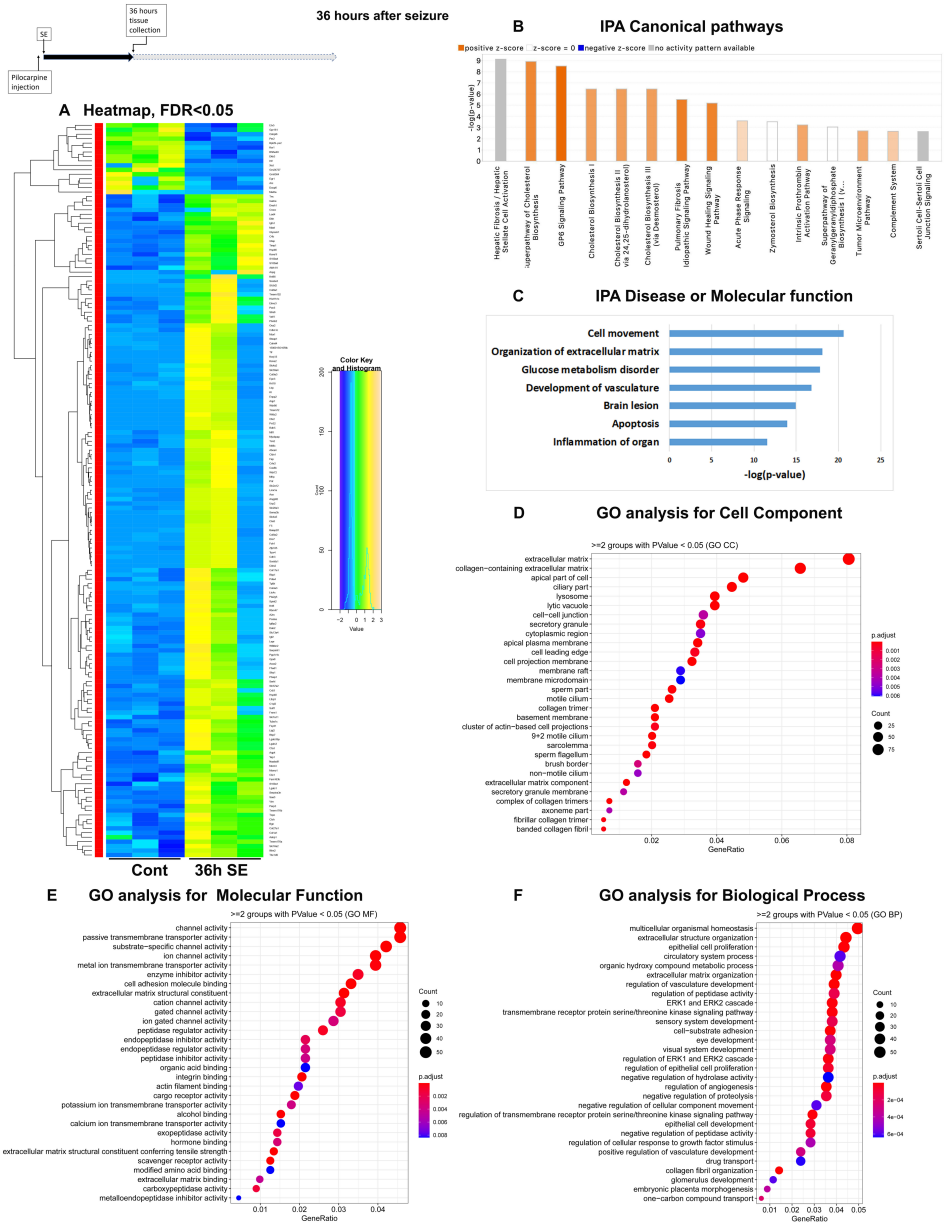
mRNA-seq analysis of changes in gene expression in mice hippocampus 36 h after the induction of SE. **(A)** Heat map of differentially expressed genes (DEGs) between control and SE mice, with False Discovery Rate (FDR) < 0.05. **(B)** IPA Canonical Pathways that are significantly enriched for DEGs between control and SE mice, with z-score > 2.0 and FDR < 0.5. **(C)** IPA Molecular Function or Disease that are significantly enriched for DEG between control and SE mice (FDR < 0.5). **(D–F)** Gene Ontology (GO) functional enrichment analyses of DEGs between control and SE mice, categorized by Cell Component **(D)**, Molecular Function **(E)**, and Biological Process **(F)** with a significance threshold of *p* < 0.05. **(G)** KEGG pathway enrichment analysis of DEGs between control and SE mice with a significance threshold of *p* < 0.05. ClusterProfiler was used to identify significantly enriched GO or KEGG categories (*p* < 0.05), group them together based on semantic similarity, and visualize the results using a dotplot. The color scale represents the adjusted *p*-value, and the dot size represents the gene count in each term.

**Figure 4:**
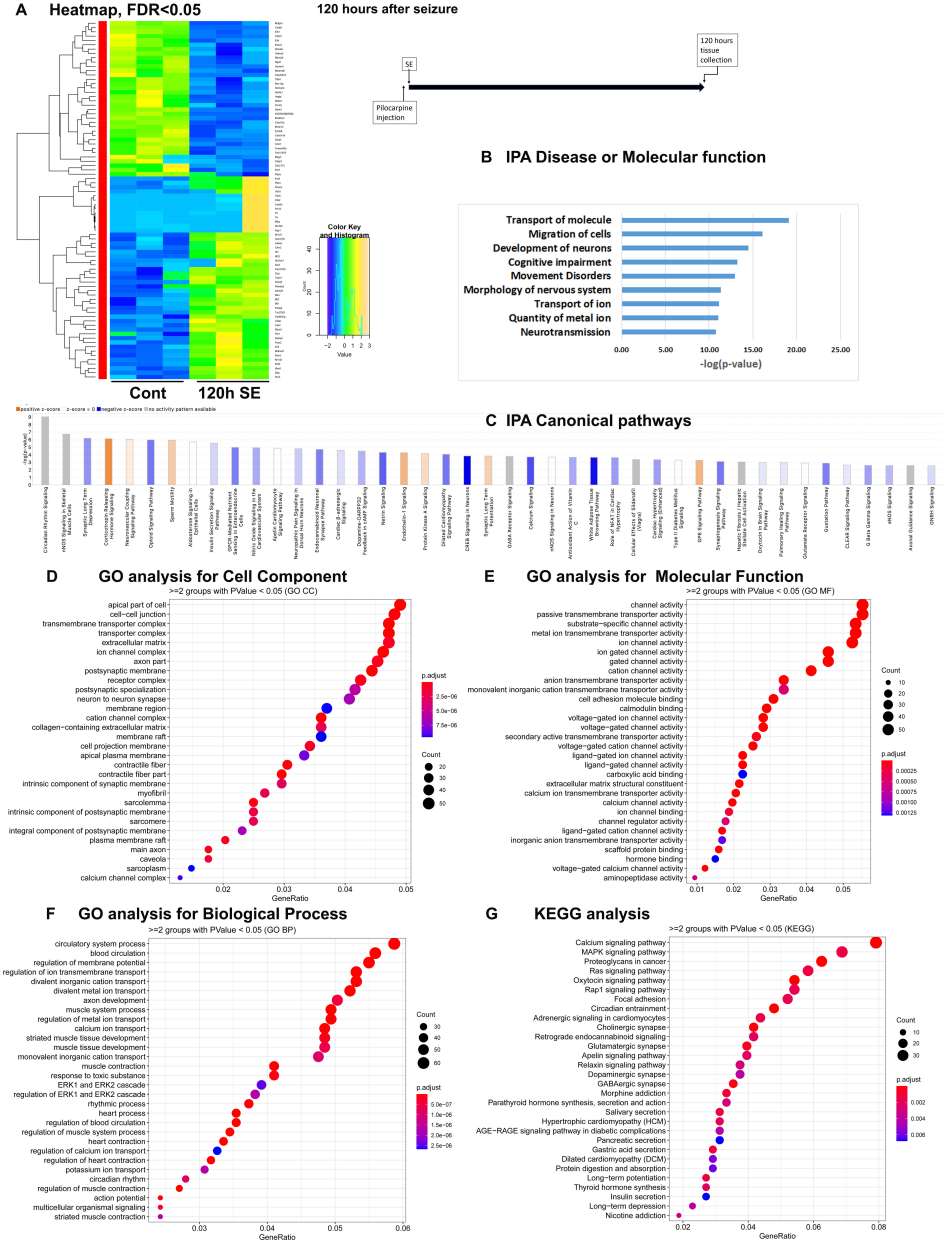
mRNA-seq analysis of changes in gene expression in mice hippocampus 120 h after the induction of SE. **(A)** Heat map of differentially expressed genes (DEGs) between control and SE mice, with False Discovery Rate (FDR) < 0.05. **(B)** IPA Canonical Pathways that are significantly enriched for DEGs between control and SE mice, with z-score > 2.0 and FDR < 0.5. **(C)** IPA Molecular Function or Disease that are significantly enriched for DEG between control and SE mice (FDR < 0.5). **(D–F)** Gene Ontology (GO) functional enrichment analyses of DEGs between control and SE mice, categorized by Cell Component **(D)**, Molecular Function **(E)**, and Biological Process **(F)** with a significance threshold of *p* < 0.05. **(G)** KEGG pathway enrichment analysis of DEGs between control and SE mice with a significance threshold of *p* < 0.05. ClusterProfiler was used to identify significantly enriched GO or KEGG categories (*p* < 0.05), group them together based on semantic similarity, and visualize the results using a dotplot. The color scale represents the adjusted *p*-value, and the dot size represents the gene count in each term.

To assess temporal alterations in the mouse hippocampus transcriptome after pilocarpine-induced seizures, we carried out mRNA-seq of hippocampus tissue isolated at four time points: 1 h, 8 h, 36 h and 5 days (120 h) after the start of pilocarpine-induced seizures in C57BL/6 J mice (Supplementary Tables S1, S3). These time points were selected to investigate alterations in physiological processes and pathways immediately after seizure initiation (1 h), after the status epilepticus (SE) phase (8 h), during the middle of latent period (36 h), and at the end of latent period (120 h) before spontaneous and recurrent seizures occur. For each time point, we mapped reads to approximately 20,000 genes. With a significance cutoff of *q* < 0.05, we identified changes in 99 differentially expressed genes (DEGs) at 1 h, with 77 genes upregulated and 22 genes downregulated (Figure 1A). At the 8-h time point, the proportion of upregulated and downregulated genes were reversed, with 59 genes showing altered expression levels, including 16 upregulated and 43 downregulated genes (Figure 2A). At the 36-h time point, the proportion of upregulated and downregulated genes was reversed again, with 165 genes showing altered expression levels, including 149 upregulated and only 16 downregulated genes (Figure 3A). Finally, at 120 h after seizure induction, we found 83 genes with altered expression, of which 47 were upregulated and 36 were downregulated (Figure 4A). For functional enrichment analysis of the mRNA-seq data (Supplementary Table S3), we used more a relaxed significance cutoff: Ingenuity Pathway Analysis (IPA) with a *q*-value <0.5 and Volcano plots (Supplementary Figure S1), DAVID/GO analysis and KEGG analysis with a *p*-value <0.05.

### Acute phase, 1 h after pilocarpine seizure induction

The group of immediate early response (IER) genes, including *Ier2, 3, Fos, Fosb, Egr1-4*, *Arc*, *Junb, Npas4, Gadd45b* and *Gadd45g* were found to be upregulated among the earliest DEG (Supplementary Figure 1A). Ingenuity Pathway Analysis (IPA) analysis revealed several Canonical Pathways (Figure 1B), including dysregulation of IGF1 Signaling, Circadian Rhythm Signaling, the CDK5 Signal Pathway (critical for regulation of migration, memory, microtubule, and cell cycle suppression), and the PKA/ERK/MAPK pathway. There were also changes in Iron Homeostasis. Disease or Molecular Functions Analysis of IPA showed alterations in Epileptic Seizure, Quantity of Cells, Expression of RNA, Quantity of Connective Tissue, and Apoptosis (Figure 1C). Gene Ontology (GO) analysis of Cellular Components identified dysregulations of Cellular Plasma Membrane and Extracellular Matrix (Figure 1D). The GO analysis of Molecular Function and Biological Processes demonstrated changes in Transcription Machinery, RNA-PolII, and MAPK Activities (Figure 1E) as well as Regulation of the ERK Cascade and Apoptotic Process (Figure 1F). KEGG analysis also identified the MAPK Signaling Pathway as the main response to seizure stress at the 1-h time point (Figure 1G).

In conclusion, our findings indicate that hippocampal cells responded to stress within one hour of the onset of seizures by upregulating the transcription of immediately early genes, genes in a group of cytokine-regulated signal pathways (IGF-1, ERK, and MAPK), as well as the RNA-PolII/transcription pathway with a significant impact on extracellular matrix.

### After status epilepticus, 8 h after pilocarpine seizure induction

The most significant changes in IPA Canonical Pathways altered were: (1). downregulation of Oxidative Phosphorylation and Mitochondrial Dysfunction; (2) Sirtuin Signaling Pathway, which regulates mitochondria physiology, Lipid Homeostasis, Apoptosis, and Oxidative Stress, (3) EIF2 Signaling, which is responsible for integrative stress response through alternations in translation and downregulation of ribosome function, and (4) upregulation of Acute Phase Response/Inflammation (Figure 2B; Supplementary Figure 1B). The top Disease or Molecular Functions affected were Movement and Mitochondrial Disorders, Glucose Metabolism Disorder, Expression of Proteins, and Immune-mediated Inflammatory Disease (Figure 2C). GO analysis revealed that the most affected Cellular Components were Mitochondria, Ribosomes, and Synapses (Figure 2D), with notable changes in Function of Membrane Transport, Ribosome Structure, Electron Transport, and Oxidoreductases Activity (Figure 2E), as well as Ribosome and Energy Metabolic Process (Figure 2F), confirming the IPA results. KEGG analysis also pointed to changes in Ribosome and Oxidative Phosphorylation (Figure 2G).

Overall, gene expression alterations observed at 8 h after pilocarpine seizure induction were characterized by increased oxidative stress, an overall down regulation of transcription, and notable changes in expression in genes related to mitochondrial structure and function, initiation of stress response through regulation of ribosome and translation/EIF2 signaling, and upregulation inflammation response.

### Beginning of the latent period, 36 h after pilocarpine seizure induction

The Canonical Pathways that underwent the most significant changes were Cholesterol Biosynthesis and GP6 (glycoprotein 6) Signaling Pathway, both of which are related to extracellular matrix (ECM) and plasma membrane. Additionally, two pathways associated with inflammation, namely the Complement System and Acute Phase Response, were upregulated (Figure 3B; Supplementary Figure 1C). Furthermore, alterations were observed in BMP Signaling Pathways. The top Diseases or Molecular Functions affected were Cell Movement, Collagen Disease, Inflammation, Apoptosis, and Transport of Molecules (Figure 3C). GO analysis revealed that the affected Cellular Components were the ECM and its components (Figure 3D). Changes in Molecular Function were related to channels, transmembrane transport, and ECM components, while changes in Biological Processes were associated with homeostasis, ECM and ERK cascade (Figure 3F).

Taken together, the results suggest a significant reorganization of neural cells, characterized by upregulation of membrane components, channels and ECM, as well as a robust inflammatory response.

### Latent period, 120 h after pilocarpine seizure induction

The IPA Canonical Pathways Analysis revealed that the top affected pathways included NOS Signaling, Synaptic Long-Term Depression and Potentiation, Corticotropin Releasing Hormone, Opioid Signaling, GABA Receptor Signaling, and Calcium Signaling Pathways (Figure 4C; Supplementary Figure 1D). The major categories detected by Disease and Functions Analysis were Migration of Cells, Transport of Molecule/Ion, Development of Neurons and Neurotransmission (Figure 4B). The GO analysis for Cellular Components showed significant alterations in Cell Membranes, Transmembrane Transporters, Ion Channels, and Synapses (Figure 4D). The GO analysis for Molecular Functions and Biological Processes revealed significant changes in Channel Activity (Figure 4E) and Membrane Potential and Ion Transport (Figure 4F), respectively. KEGG analysis demonstrated dysregulation in Calcium and MAPK Signaling Pathways (Figure 4G).

Therefore, during the latent period following pilocarpine seizures, the gene expression differences were primarily observed in genes associated with neuronal function, specifically ion transport, membrane channels, and synapses.

### MicroRNA-seq

To assess temporal changes in small RNA profiles in the mouse hippocampus following seizures, we carried out microRNA-seq of the hippocampus tissue samples isolated from groups of C57BL/6 J mice at specific time points, which were mostly consistent with those used for the previously described mRNA-seq analysis. The time points included 1 h, 8 h, 24 h (different from the 36-h time point of the mRNA-seq analysis) or 5 days (120 h) after the initiation of pilocarpine-induced seizures. Our analysis revealed the presence of not only miRNA, but also piRNA and snoRNA in the hippocampal samples (Supplementary Table S4). Small nucleolar RNAs (snoRNAs) are a class of ancient small non-coding RNAs that play a fundamental role in the modification and processing of ribosomal RNA (Scott and Ono, 2011). Piwi-interacting RNAs (piRNAs) are a recently identified class of small non-coding RNAs which direct the Piwi-dependent transposon silencing, heterochromatin modification, and germ cell maintenance, and have also been detected in neuron-enriched exosomes (Quek et al., 2017).

**Figure 5:**
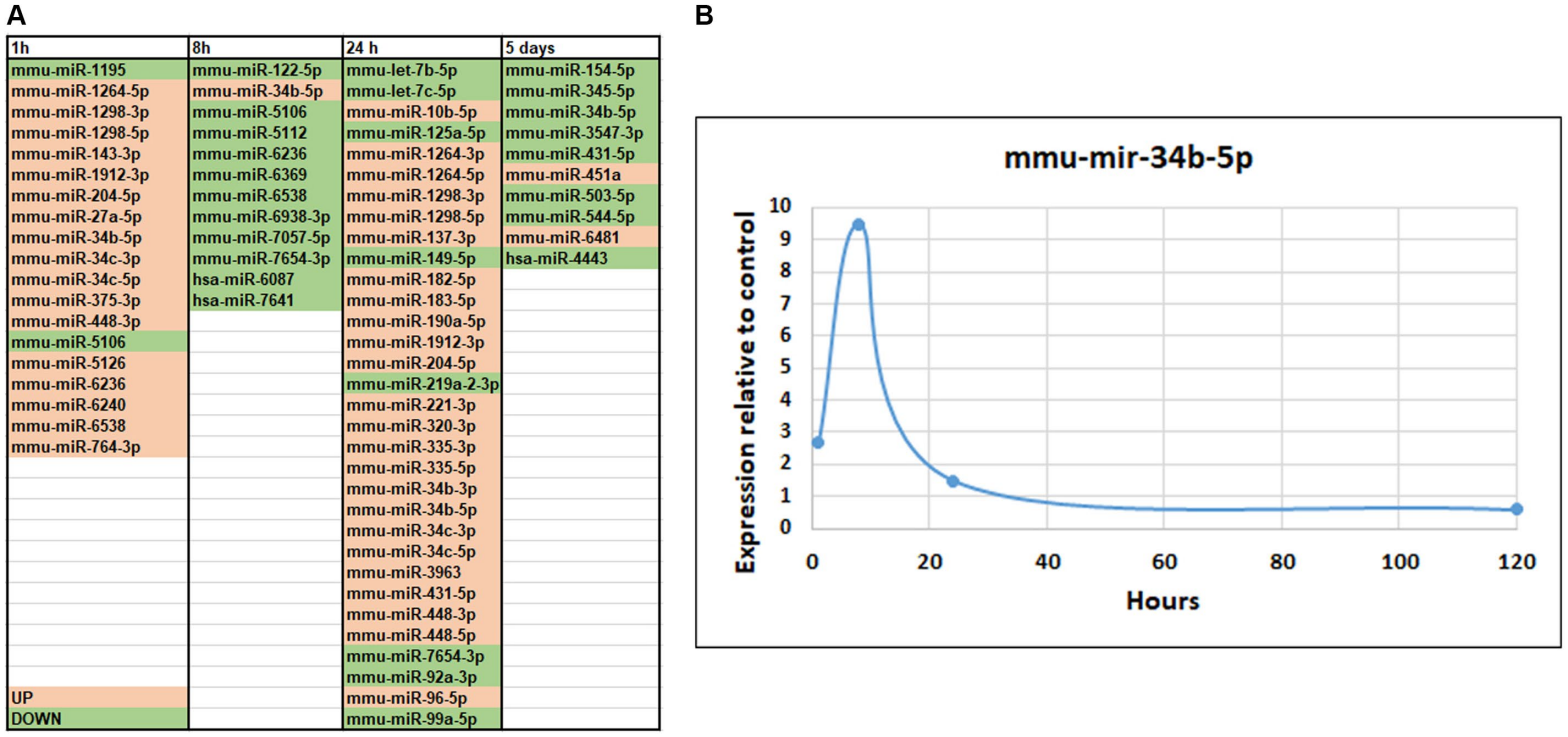
MicroRNA-seq analysis of changes in small RNA expression in mice hippocampus after SE. **(A)** List of differentially expressed microRNAs. The genes of which expression was altered are shown in pink (up-regulated) or green (down-regulated). **(B)** Temporal changes in *miR-34b-5p* after SE demonstrating the maximum change at 8-h time point.

We observed differential expression of several piRNAs and/or piRNA-like molecules (Koduru et al., 2018). Notably, it has been reported by other researchers that the orthologues of PIWI-proteins are expressed in the mammalian brain (Kim, 2019). We identified small RNA genes that exhibited differential expressions between control and seizure groups, with a significance level of *p* < 0.05, as shown in Figure 5A. At the 1-h time point, we observed upregulation of 17 microRNAs and downregulation of only 2 microRNAs (Figure 5A). Among the upregulated microRNAs, several have been previously reported to exhibit differential expression in patients with epilepsy (*miR-1298, 375, 34c*) (Organista-Juárez et al., 2019; Fu et al., 2020) or traumatic brain injury (*miR-27a*) (Sabirzhanov et al., 2014). *MiR-143* has been implicated in increasing blood–brain barrier permeability (Bai et al., 2016, 2018) while *miR-1912, 204* (Kaalund et al., 2014; Chiu et al., 2019) and *34b* have been associated with pro-apoptotic effects (Liu et al., 2016).

At 8 h after pilocarpine injection, we observed downregulation of 11 microRNA and upregulation of only 1 microRNA (Figure 5A). *MiR-122* has been implicated in ischemia (Guo et al., 2018). At 24 h, 25 microRNAs exhibited upregulated expression, while only 8 displayed downregulated expression (Figure 5A). Notably*, let-7b* and *let-7c*, which were downregulated, have previously been associated with brain injuries and seizures and suppression of apoptosis (Ni et al., 2015; Han et al., 2018). Conversely, *miR-125* has been linked to induction of apoptosis (Yao et al., 2021). At 120 h, 8 microRNAs were downregulated, with only 2 showing upregulated expression (Figure 5A). It is noteworthy that downregulated microRNA miR-154-5p has been implicated in the regulation of synaptic plasticity (Rahmani et al., 2022) and seizures (Zhao et al., 2020), while miR-503-5p has been implicated in apoptosis (Tang et al., 2019).

### Temporal changes in biological pathways

**Figure 6:**
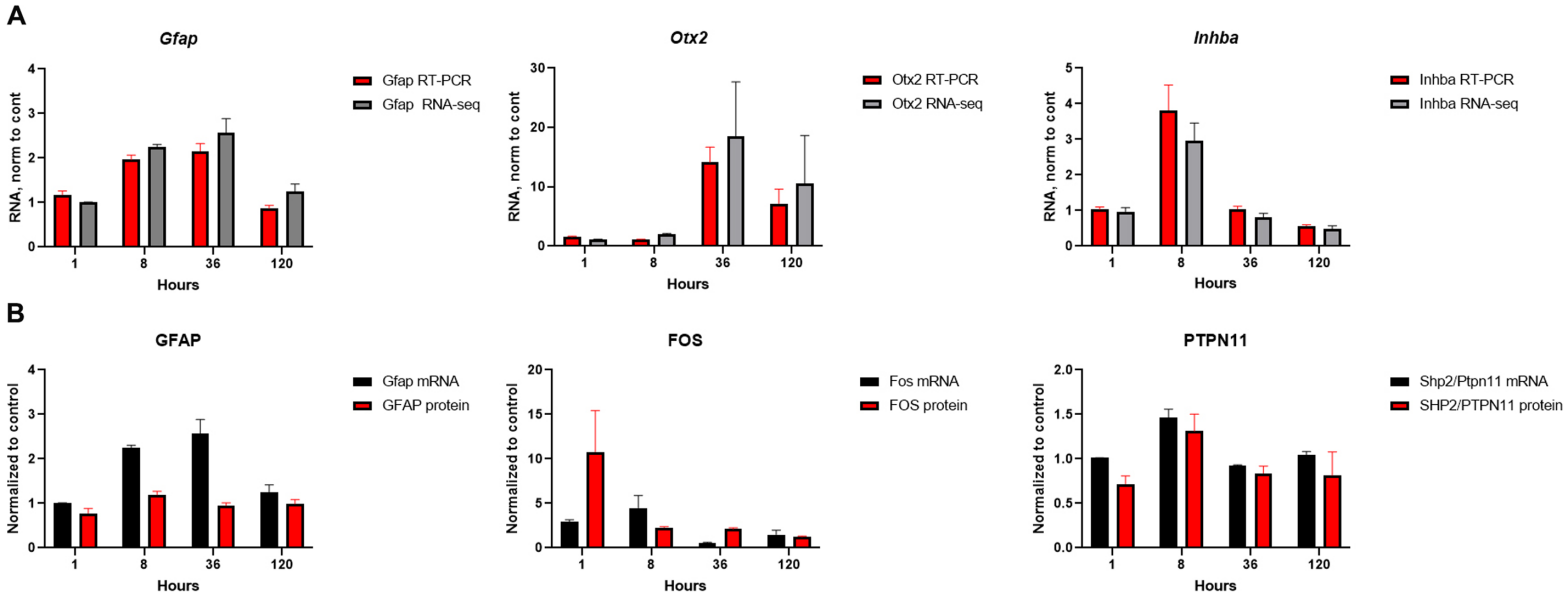
Validation of mRNA-seq results. **(A)** Comparison of mRNA-seq results with qRT-PCR for selected genes; RNA amount at SE samples was normalized to the amount of RNA in control samples. **(B)** Comparison of mRNA-seq results for RNA expression with protein expression by western blot for selected genes/proteins; β-actin was used as the loading control. The RNA and protein amounts in SE samples were normalized to amount of RNA and protein in control samples. Experiments were conducted with two to four biological and three technical replicates.

We conducted a comprehensive investigation of the temporal dynamics of gene expression changed in order to gain insights into the optimal time points for potential therapeutic interventions. Our conclusion about temporal alterations in gene expression and pathways were made from mRNA-seq data and extensive analysis of these data by different approaches. Analysis of several genes (Gfap, Otx2, Inhba) and proteins (GFAP, FOS, PTPN11) expression by qRT-PCR and Western blotting were carried out for validation and demonstrated that mRNA-seq data corroborates with other biochemical methods (Figures 6A,B; Supplementary Figure S1).

**Figure 7:**
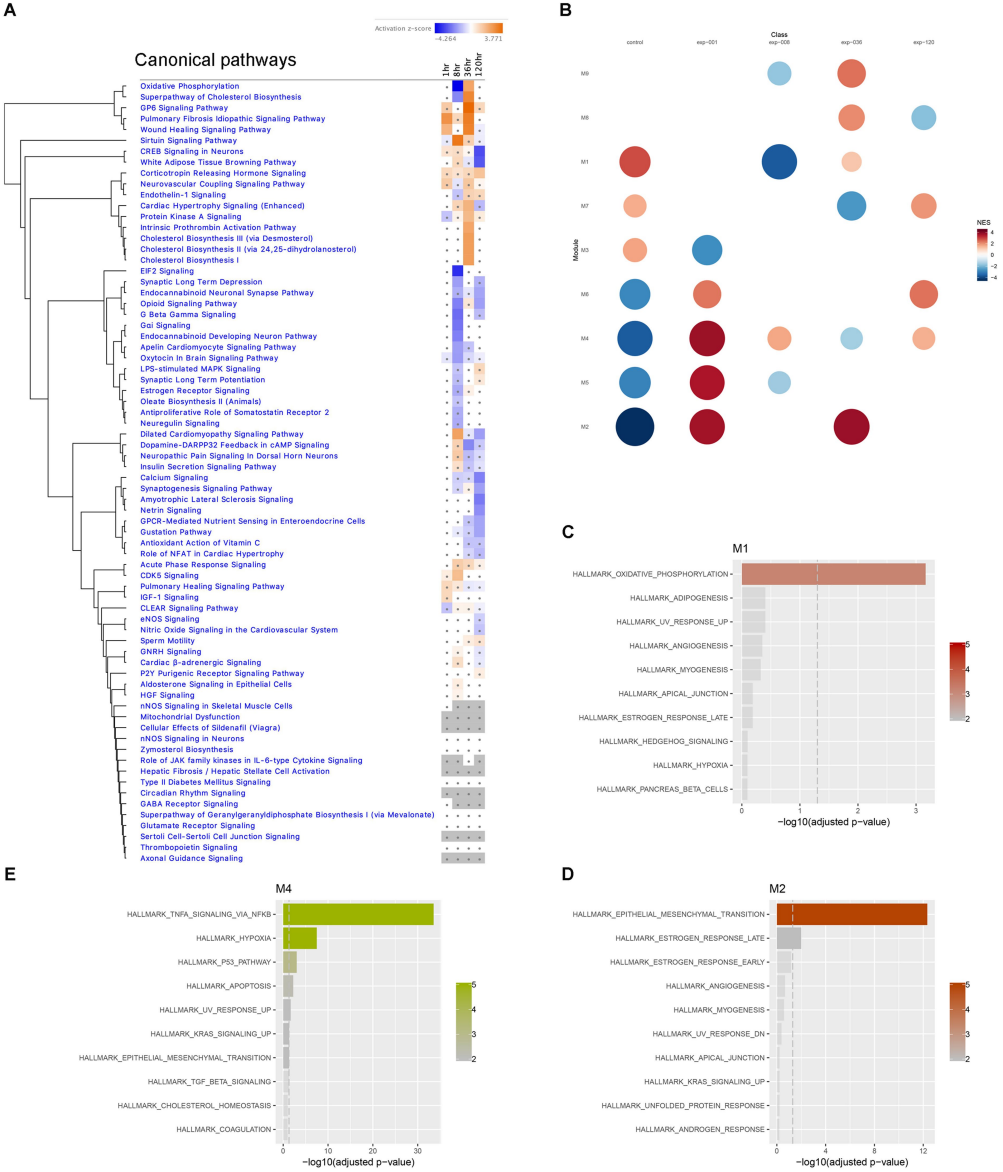
Temporal transcriptome dynamics in hippocampus. **(A)** Temporal heatmap depicting the direction of change in gene expression for the IPA Canonical Pathways. Color intensities indicate the level of changes (activation *z*-score), with blue indicating inhibition and orange indicating activation. Asterisks indicate significant changes. **(B)** Gene set enrichment analysis of each module yielded Normalized Enrichment Score (NES) of modules, with red represents higher activity and blue represents lower activity, and the color intensity and the size of the dots represents significance levels. **(C–E)** Biological functions associated with each module was determined by Over Representation Analysis of CEMiTool for modules M1, M2 and M4, respectively.

First, we plotted a heatmap of the Canonical Pathway changes from IPA analysis in a time series, which revealed significant clustering of pathways with altered activation Z-scores. Notably, Oxidative Phosphorylation and Superpathway of Cholesterol Biosynthesis exhibited a drastic suppression at the 8-h time point, followed by a reversal to activation at the 36-h time point (Figure 7A). Similarly, CREB Signaling Pathway showed reversed activation to suppression between 36-and 120-h time points, while Synaptic Long Term Potentiation was suppressed at 8-h and then reversed to activation at 120-h time point (Figure 7A). These findings suggest the presence of dynamic temporal ‘switches’ in the molecular makeup of the hippocampus during epileptogenesis, while certain pathways, such as Corticotropin Releasing Hormone Signaling, consistently showed activation throughout the time course (Figure 7A). Additionally, we performed module co-expression analyses using the CEMiTool package (Russo et al., 2018) and identified 9 distinct co-expression modules (Figure 7B), out of which 3 were significantly enriched with pathways in Over Representation Analysis (Figures 7C,D); Module M1 was significantly enriched with Oxidative Phosphorylation Pathway (Figure 7C), module M2 showed enrichment in Epithelial-Mesenchymal Transition (EMT) and Estrogen Response Pathways (Figure 7D), and module M4 was enriched in TNFA Signaling via NFKB, Hypoxia, P53 Pathway, and Apoptosis Pathways (Figure 7E). Subsequently, we further investigated these prominent biological pathways and functions in greater detail.

The immediate early response (IER) gene set, including *Fos* (Figure 6B; Supplementary Figure S1)*, Fosb, Arc, Erg3*, showed significant upregulation during the acute stage (1-h time point), and some of these genes remained upregulated at 8 h, suggesting their potential involvement in triggering later changes in metabolic pathways in the hippocampus. However, several of IER genes (*Arc, Egr1, Egr2*) were downregulated at 36 h, while *Gadd45g* continued to show upregulation at both 36- and 120-h time points. Notably, all IER genes are present in module M4, which was enriched in TNFA signaling via NF-kB (Figure 7E).

**Figure 8:**
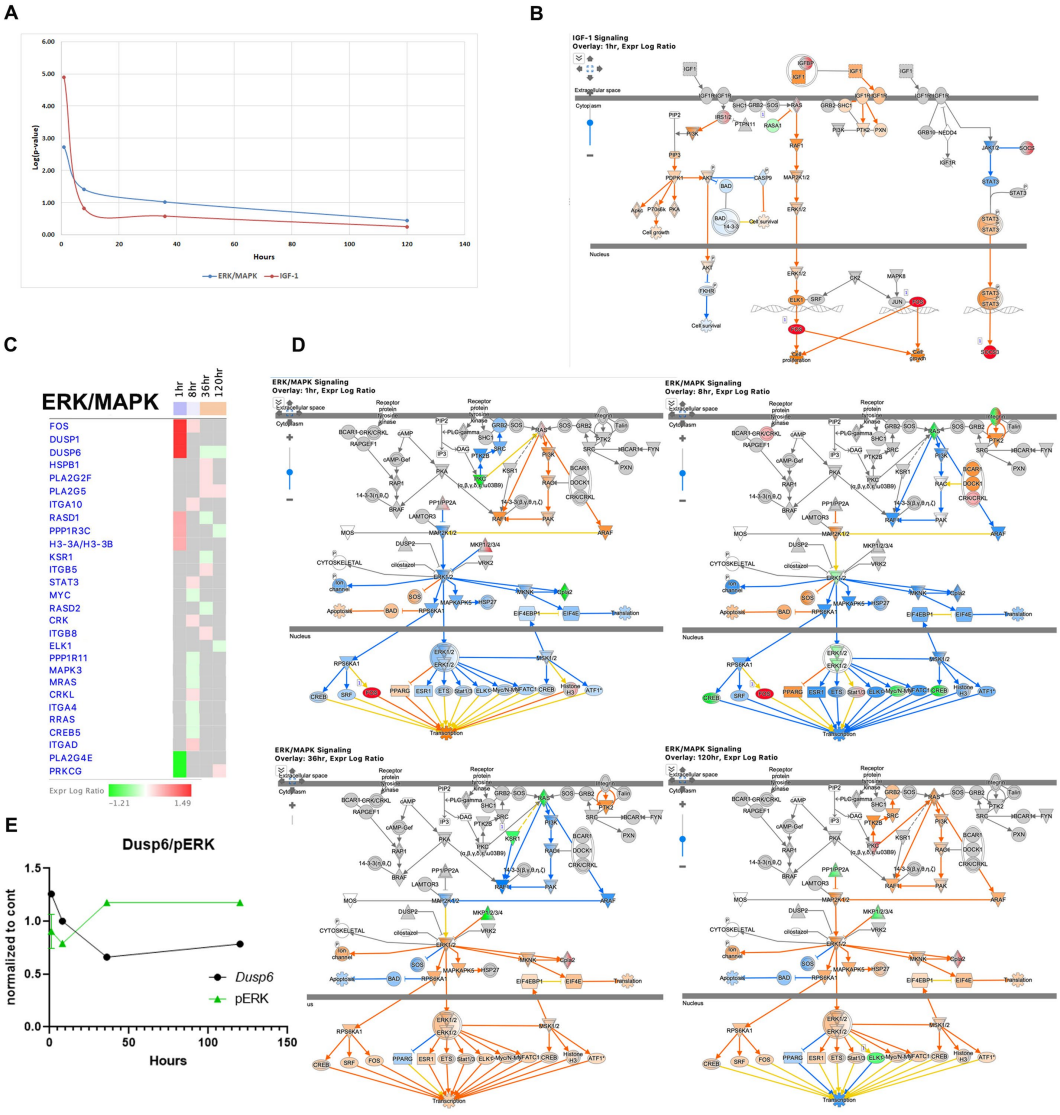
Temporal molecular changes in hippocampus after SE in IGF-1 and ERK/MAPK signaling pathways. **(A)** Both pathways exhibit drastic changes immediately after the onset of SE, followed by a rapid return to baseline levels. Y-axis on graph is -Log (*p*-value), where *p*-value is for significance of pathway at particular timepoint according to IPA analysis **(B)**. IPA Canonical Pathway, “IGF-1 Signaling Pathway” at 1 h after the onset of SE. The nodes represent genes/molecules/complexes in a pathway, and the lines and arrows between nodes indicate known relationships from the Ingenuity Knowledge Base. Nodes with the intensity of colored infill indicate the level of up (red) or down (green) regulated genes in SE relative to control. The blue- (inhibition) and orange- (activation) colored molecules and lines are predicted activation states generated by IPA. Yellow lines indicate relationships where our findings are inconsistent with the state of the downstream molecule. The molecules in the pathway are given shapes that indicate their functional class (Nested Circle/Square = Group/Complex, Horizontal ellipse = Transcriptional Regulator, Vertical Ellipse = transmembrane receptor, Vertical Rhombus = enzyme, Square = Cytokine/Growth Factor, Triangle = Kinase, Vertical Ellipse = Transmembrane Receptor, Circle = other). The edges between molecules are also differentiated to indicate the type of relationship between them. Solid lines are direct relationships and dashed lines are indirect. **(C)** Temporal heatmap depicting the direction of change in gene expression for the ERK/MAPK Signaling Pathway. The colored boxes beneath each time point label indicate the overall direction of pathway activation at that specific time point, with blue indicating inhibition and orange indicating activation. **(D)** IPA Canonical Pathway, “ERK/MAPK Signaling Pathway” at 1, 8, 36 and 120 h after the onset of SE. **(E)** Temporal relation between dual-specificity phosphatase 6 (*Dusp6*) mRNA and phosphorylated protein ERK.

The IGF1 pathway is known to enhance hippocampal excitatory and seizure activity and to activate the ERK/MAPK pathways, exhibited immediate upregulation during acute seizure phase (1-h time point) with increased expression of genes such as *Ccn1/Cyr61, Ccn2/Ctgf, Fos, Igfbp2, Irs2, Rasa1, Rasd1, and Socs3.* However, this pathway is less pronounced at later stages (Figures 8A,B). The ERK/MAPK pathway, implicated in SE, seizure, and epilepsy (Hansen et al., 2014; Gangarossa et al., 2015; Ahmed et al., 2021) showed early dysregulation during the acute phase (Figures 8C,D), which genes such as dual-specificity phosphatase 6 (*Dusp6*), specific for ERK1/2 (Queipo et al., 2017), being upregulated at the 1-h time point, but then down regulated at 36 and 120 h. Consistent with this finding, the level of phosphorylated ERK showed an inverse pattern, with a decrease at 8 h followed by an increase at 36 h (Figure 8E). Overall, the ERK/MAPK pathway showed the most dysregulation at the 1-h time point and slight upregulation at the 36- and 120-h time points (Figures 8A,C,D).

**Figure 9:**
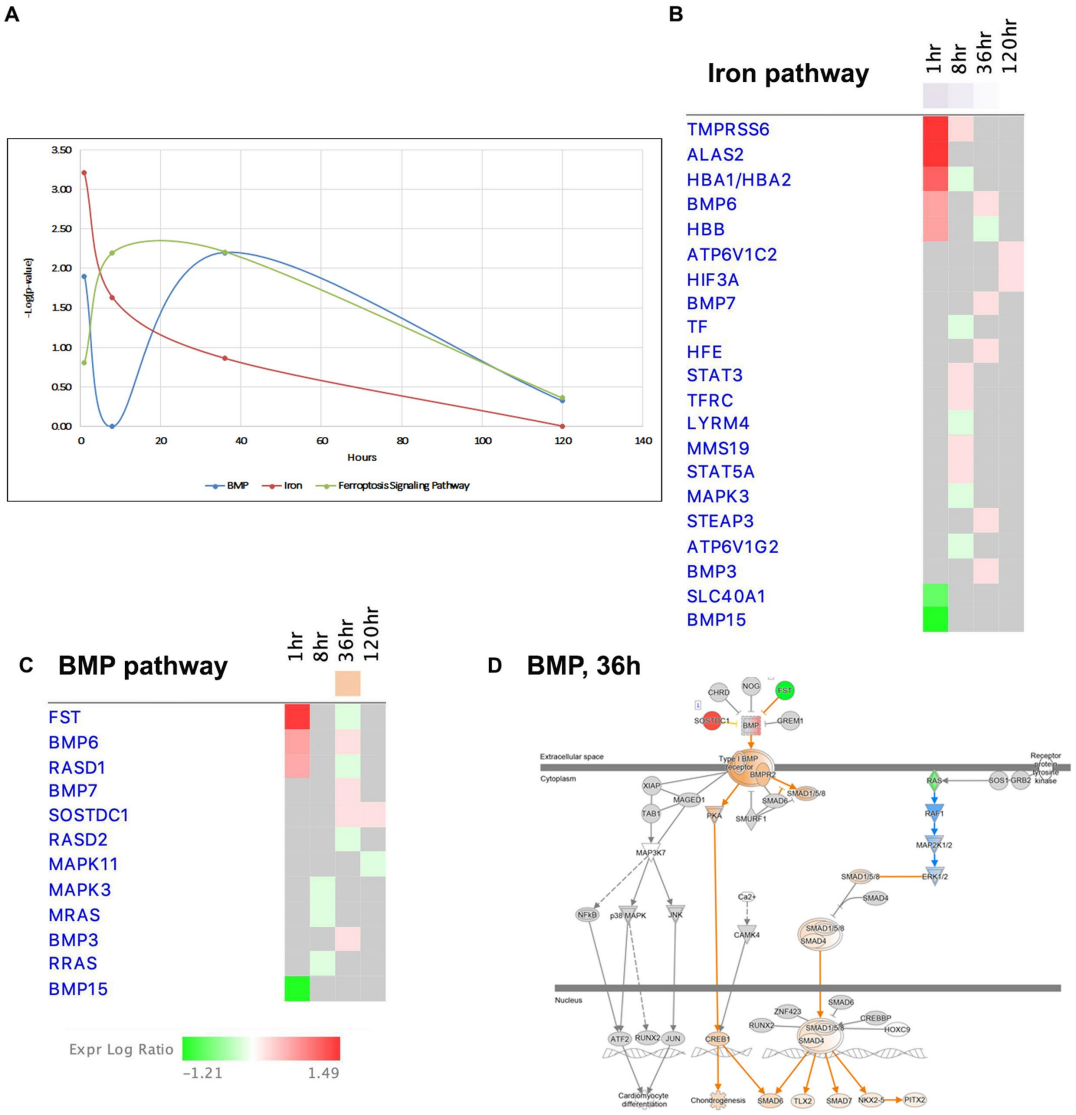
Temporal molecular changes in hippocampus after SE in BMP/Iron related pathways. **(A)** Temporal changes in BMP, Iron, and Ferroptosis Signaling Pathways. Y-axis on graph is-Log (*p*-value), where *p*-value is for significance of pathway at particular timepoint according to IPA analysis. **(B)** Temporal heatmap depicting the direction of change in gene expression for the Iron Pathway. **(C)** Temporal heatmap depicting the direction of change in gene expression for the BMP Pathway. **(D)** IPA Canonical Pathway, “BMP Pathway” at 36 h after the onset of SE.

The Iron signaling pathway exhibited dysregulation, as evidenced by altered expression of various genes (Figures 9A,B). For example, the transferrin gene (*Trf*/TF) was downregulated at 8 h, while the transferrin receptor gene *Tfrc* was upregulated at same time. In addition, the inhibitor of iron uptake gene *Hspb1* and homeostatic iron regulator gene *Hfe* were upregulated at 36 h. Genes involved in intracellular iron trafficking such as *Steap1-4* (36 h) and *Alas2* (1 h) were also upregulated. Conversely, the gene for the channel *Slc40a1*, which exports iron from cells, was downregulated at 1 h. Despite ion’s potential involvement in lipid peroxidation, we observed downregulation of genes encoding enzymes associated with this process, including *Acsl4* (120 h), *Alox12e* (8 h), and *Alox12b* (36 h).

Recent findings have suggested that bone morphogenic proteins (BMP) may regulate iron homeostasis in non-neuronal tissues (Muckenthaler et al., 2017; Xiao et al., 2020), and BMP proteins are known to participate in neurogenesis and exhibit neuroprotective properties. Interestingly, the BMP pathway, which shares many common genes with the iron signaling pathway (Figures 9A,C,D), displayed dysregulation at 1 h and 36 h, but transiently returned to the control level (i.e., no dysregulation) at 8 h (Figure 9D). Based on these observations, we propose that the iron regulation/BMP pathways may undergo significant changes during epileptogenesis and could potentially serve as novel targets for seizure treatment.

Ferroptosis, a recently discovered pathway that regulates cell death, is characterized by intracellular accumulation of iron, leading to lipid peroxidation and increased production of reactive oxygen species (ROS) (Mao et al., 2019a). Notably, we observed changes in the ferroptosis signaling pathway at 8 and 36 h (Figure 9A). Previous studies have suggested that cells in the hippocampus may be protected from ferroptosis by glutathione-mediated detoxification (Mao et al., 2019b; Zimmer et al., 2021). Our data demonstrated upregulation genes involved in glutathione metabolism pathway, such as Cd*44, Slc7a11, Gpx3* and *Gpx8,* at the 36-h time point.

**Figure 10:**
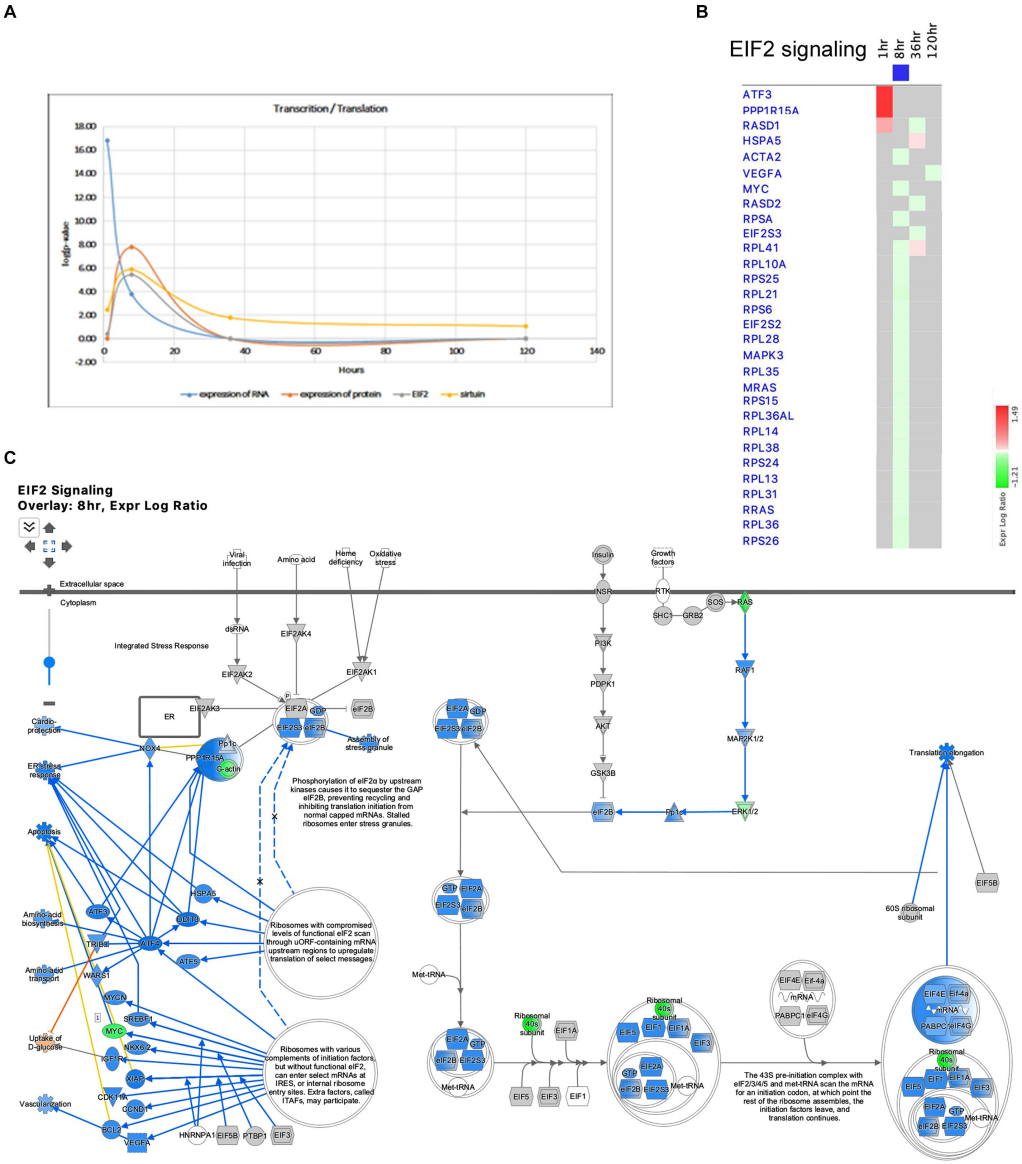
Temporal molecular changes in hippocampus after SE in the pathways related to transcription and translation. **(A)** Temporal changes in expression of RNA, expression of protein, EIF2 Signaling, and Sirtuin Pathways demonstrating consistent changes at 8-h time point. Y-axis on graph is-Log (*p*-value), where *p*-value is for significance of pathway at particular timepoint according to IPA analysis. **(B)** Temporal heatmap depicting the direction of change in gene expression for the EIF2 Signaling Pathway. **(C)** IPA Canonical Pathway, “EIF2 Signaling Pathway” at 8 h after the onset of SE.

The Pathways and Molecular Functions analysis of the IPA revealed the dysregulation of pathways related to transcription and translation after SE. The transcriptional response to seizure showed changes in RNA polymerase II binding as early as 1 h after SE (Figure 1E), and the Molecular Function of Expression of RNA exhibited the most significant changes at that time point (Figure 10A). The EIF2 pathway, which plays a crucial role in integrated stress responses, regulation of translation, and ribosome function, was observed to be altered during the initial stages following pilocarpine injection, particularly at 8 h (Figures 2C, 10A–C). At this time point, 18 ribosomal genes were downregulated (Figures 10B,C). Furthermore, the downregulation of the beta subunit of the eIF2 complex (*Eif2s2*) at 8 h could lead to the inhibition of general translation and increased translation of specific mRNAs with short inhibitory upstream open reading frames (uORF) (Figure 9C), such as the transcriptional activator *Cebpa* (upregulated at 8 h) and the gene involved in the EIF2 pathway *Ppp1r15a* (upregulated at 1 h). Additionally, another gene involved in stress responses, *Ucp2*, which acts as a defender against oxidative stress in mitochondria (Hass and Barnstable, 2021) and has a known inhibitory uORF, was found to be upregulated at later time points (upregulated at 36 and 120 h).

**Figure 11:**
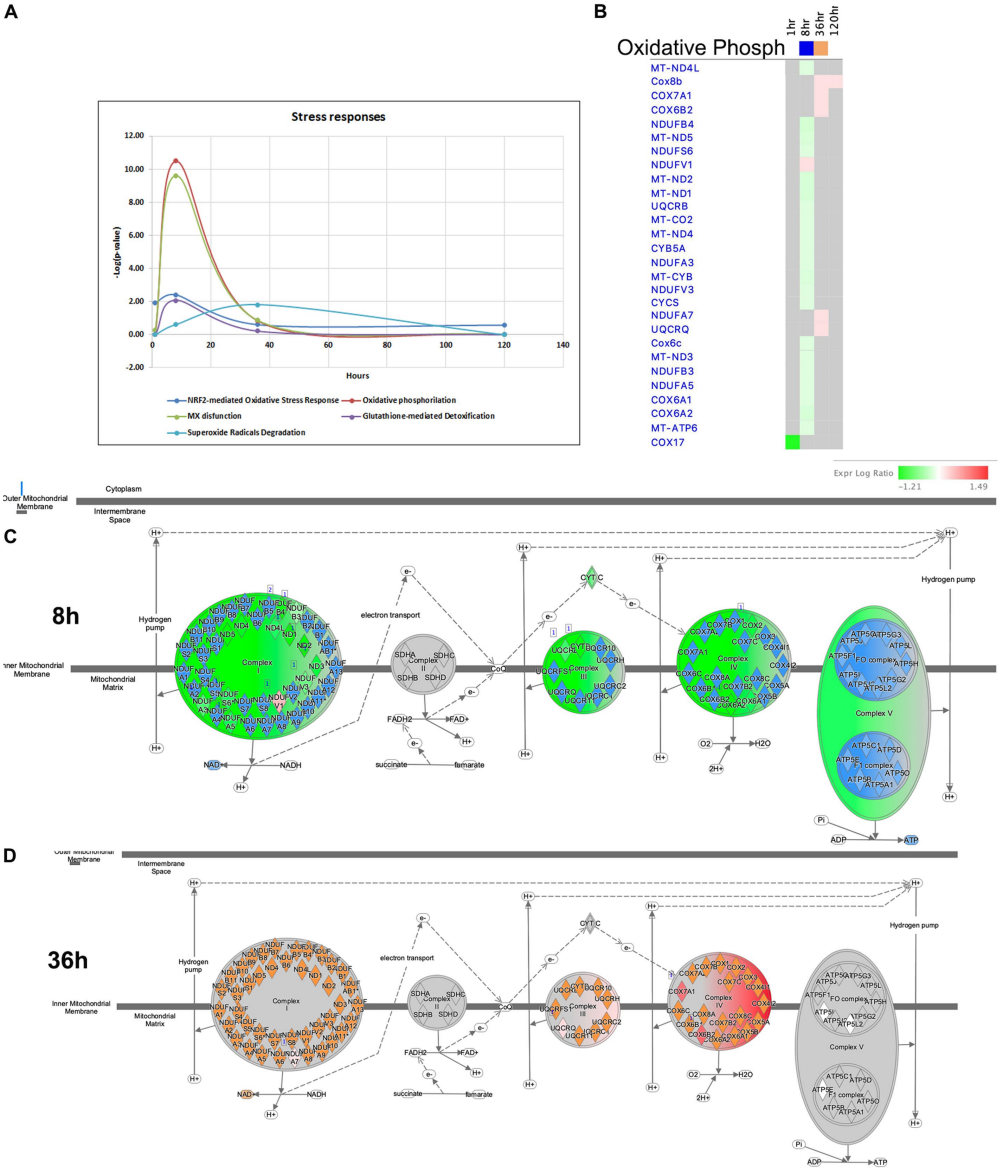
Temporal molecular changes in hippocampus after SE in stress responses related pathways. **(A)** Temporal changes in NRF2-mediated Oxidative Stress Response, Oxidative Phosphorylation, MX dysfunction, Glutathione-mediated Detoxification, and Superoxide Radicals Degradation Pathways demonstrating greater changes at 8-h time point. Y-axis on graph is-Log (*p*-value), where *p*-value is for significance of pathway at particular timepoint according to IPA analysis. **(B)** Temporal heatmap depicting the direction of change in gene expression for the Oxidative Phosphorylation Pathway. **(C,D)** IPA Canonical Pathway, “Oxidative Phosphorylation Pathway” at 8 and 36 h after the onset of SE, respectively.

It is widely recognized that oxidative stress and mitochondrial dysfunction are significant contributors to the neuropathology of seizure, epilepsy, and SE. Disruption of the delicate balance in reactive oxygen species (ROS) is a consequence of the excessive production of these radicals and impairment of antioxidant pathways (Olowe et al., 2020). The complexes of the mitochondrial respiratory chain are major sources of ROS production. Antioxidant systems, including NRF2-mediated Oxidative Stress Response, Superoxide Radical Degradation, and Glutathione-mediated Detoxification, play crucial roles in maintaining redox homeostasis (Kovac et al., 2017; Pearson-Smith and Patel, 2017). In our model of TLE, significant impairments in mitochondrial function were observed at 8 h after pilocarpine injection (Figures 7B,C, 11A–C). At this time point, multiple genes associated with Complex I, III and IV of the Mitochondrial Respiratory Chain were downregulated, leading to potential increase in oxidative stress. For instance, if the supply of NADH exceeds the capacity of Complex I to rapidly oxidize it, electrons from NADH may interact with O_2_ to form superoxide molecules, a form of ROS (Hass and Barnstable, 2021). At 36-h time point, however, several genes related to the Cytochrome Oxidase Complex were upregulated, potentially resulting in decrease of ROS production and oxidative stress (Figures 7B, 11B,D). Furthermore, genes involved in antioxidant pathways, such as NRF2-mediated Oxidative Stress Response and Glutathione-mediated Detoxification, exhibited their most significant changes at the 8-h time point (Figure 11A). Dysregulation of NRF2-mediated Oxidative Stress Response Pathways was observed, while stress-induced genes were upregulated (*Bach1, Maff, Mafg, Fkbp5*), and expression of antioxidant genes, including *Gstm1, Mgst3, Cat, Mapk3* (ERK1), and *Pmf1*, were down-regulated. The Superoxide Radical Degradation Pathway showed a maximum change at 36 h, with upregulation genes of such genes as *Sod3* and *Nqo1*. However, if the changes in the antioxidant systems were insufficient to completely mitigate mitochondrial damage (Figure 11A), oxidative stress may not be fully alleviated, potentially triggering new seizures and further damage to the hippocampus.

**Figure 12:**
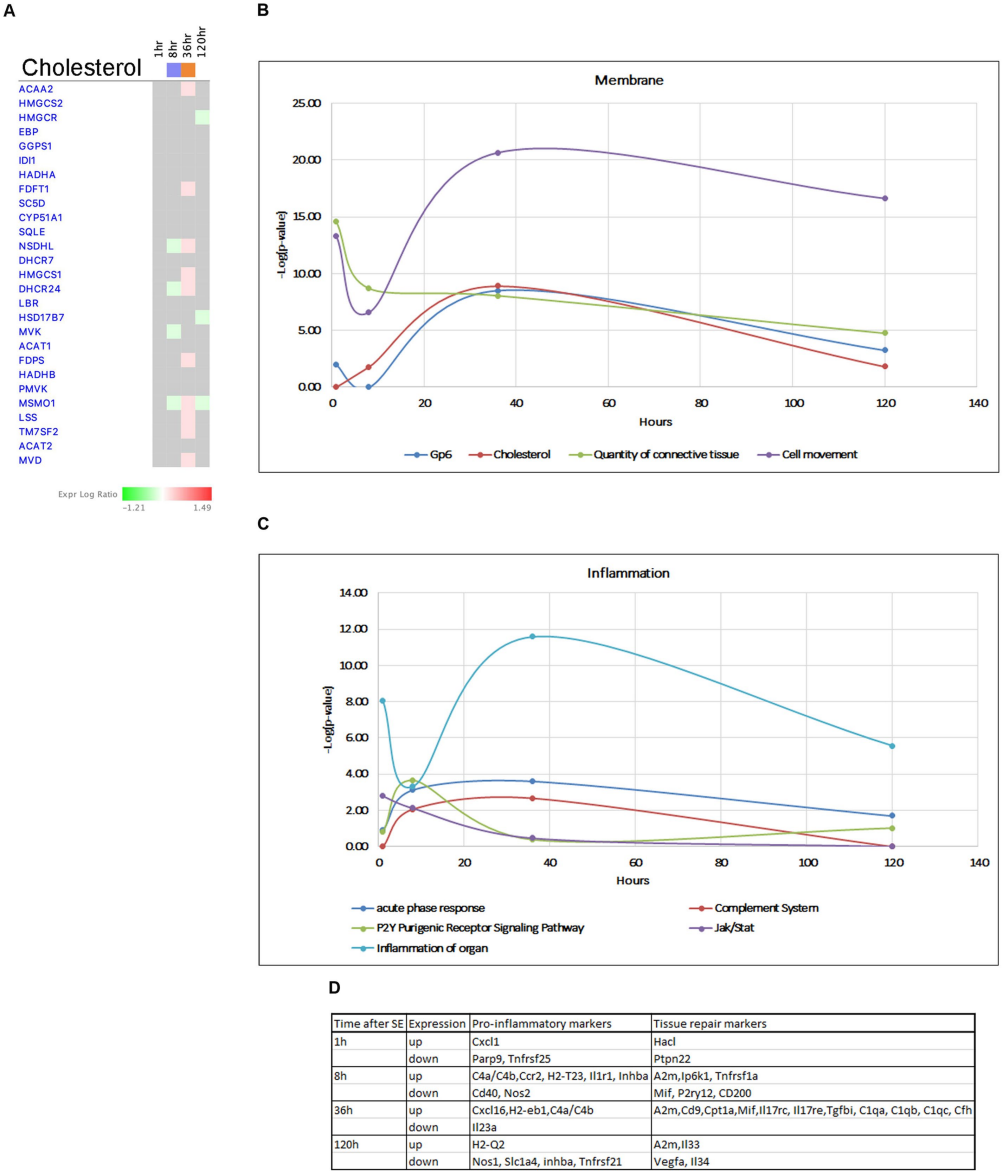
Temporal molecular changes in hippocampus after SE in cholesterol biosynthesis related pathways. **(A)** Temporal heatmap depicting the direction of change in gene expression for the Superpathway of Cholesterol Biosynthesis. **(B,C)** Temporal changes in pathways related to membrane and cell connection and inflammation, respectively. Y-axis on graphs is-Log (*p*-value), where *p*-value is for significance of pathway at particular timepoint according to IPA analysis. **(D)** Gene classification based on the module co-expression analysis.

Pathways involved in plasma membrane homeostasis, such as GP6 and Cholesterol Biosynthesis were activated at 36 h (Figures 12A,B). Cholesterol is an important structural component of myelin and cellular membranes in the brain and it is synthesized autonomously in the brain, as it cannot readily cross the blood–brain barrier (BBB) (Orth and Bellosta, 2012; Zhang and Liu, 2015). Production of cholesterol and its regulation are cell-specific in brain cells, with astrocytes being the main producers of lipids that are transported to neurons and regulate synapse development and function (van Deijk et al., 2017). Cholesterol biosynthesis starts from acetyl-CoA and involves more than 20 enzymes participating in more than 30 enzymatic reactions (Waterham, 2006; Mitsche et al., 2015). Our data showed that the Superpathway of Cholesterol Biosynthesis had strong suppression at the 8-h time point, but this suppression was reversed to activation at the 36-h time point. We observed upregulation of several genes for cholesterol biosynthesis (*Acaa2, Hmgcs1, Mvd, Fdps Fdft1, Lss, Msmo1, Nsdhl, Dhcr24, Tm7sf2*) (Figure 12A) at the 36-h time point. Additionally, we found an up-regulation of a set of genes involved in cholesterol transport within the brain at the same time point (*Lamp2, Npc2, Stard4*), with some of them continuing to be elevated at 120 h. Changes in cholesterol biosynthesis have recently been suggested in epileptogenesis (Hanin et al., 2021). We propose that changes in cholesterol metabolism are a major component of the hippocampus response to seizures, and increased cholesterol production may have neuroprotective effects.

The blood–brain barrier (BBB) serves as a physiological barrier that separates blood from brain parenchyma, and is composed of tightly connected endothelial cells with tight junctions. Seizures and epilepsy have been shown to potentially compromise the function and integrity of the BBB, leading to increased permeability. Our data indicate activation of GP6 Signaling Pathway and genes in extracellular matrix during several time points following seizure (Figure 7A), with peak activation observed at 36 h (Figure 12B). This is accompanied by upregulation of multiple collagen genes, tight junction complex genes such as claudins *Cldn2*, cingulins *Cgn, Cgnl1,* laminins *Lamb2, Lama5*, as well as *Marveld3, Tjp3*, ECM/Integrin signaling pathway genes *Spp1, Itgb5, Itgb8, Bcam,* and gap junction gene *Gja1.* These findings suggest a potential neuroprotective role for the increased expression of these genes at 36 h post-seizure. On the other hand, we also observed downregulation of two important collagen genes, *Col4a1/2* at 120 h, indicating that the tissue may become susceptible to seizures again at the end of the latent phase.

Inflammation has been recognized as a hallmark of seizures, but it remains unclear whether it is a secondary reaction to neuronal insult or it plays an active role in epileptogenesis. Additionally, the beneficial or harmful nature of inflammation and its impact on neurotoxicity remains unclear. Immune responses to seizures are typically delayed by 24–48 h (Eyo et al., 2017) from the onset of SE, and it has been suggested that activation of JAK/STAT pathway may lead to induction of inflammatory responses (Okamoto et al., 2010; Tian et al., 2017). Our data support this, as we observed changes in the JAK/STAT pathway as early as 1 h after pilocarpine injection (Figure 1B), which persisted up to 8 h (Figure 12C), with upregulation of genes such as *Stat3, Stat5, Cdkn1a,* and *Ptpn11* (Figure 6B). Furthermore, our analysis demonstrated initiation of TNFA signaling pathway at the 1-h time point (Figures 7B,E), and increased gene expression in Acute Phase Response (Figure 7A), Complement System and P2Y-purinergic Receptor Pathways, all associated with inflammation and immune response, at the 8-h time point. Some of these changes continued at 36 h after start of seizure (Figure 12C), with upregulation of genes such as *Il1r1, Tnfrsf1a, C4b, A2m, C1qa, C1qb, C1qc, Cfh, P2ry12, H2-T23, H2-Eb1,* and *Ccr2*. Overall, we observed upregulation of more proinflammatory genes at 8 h, where anti-inflammatory and tissue repair genes were upregulated at 36 h post-seizure (Figure 12D).

**Figure 13:**
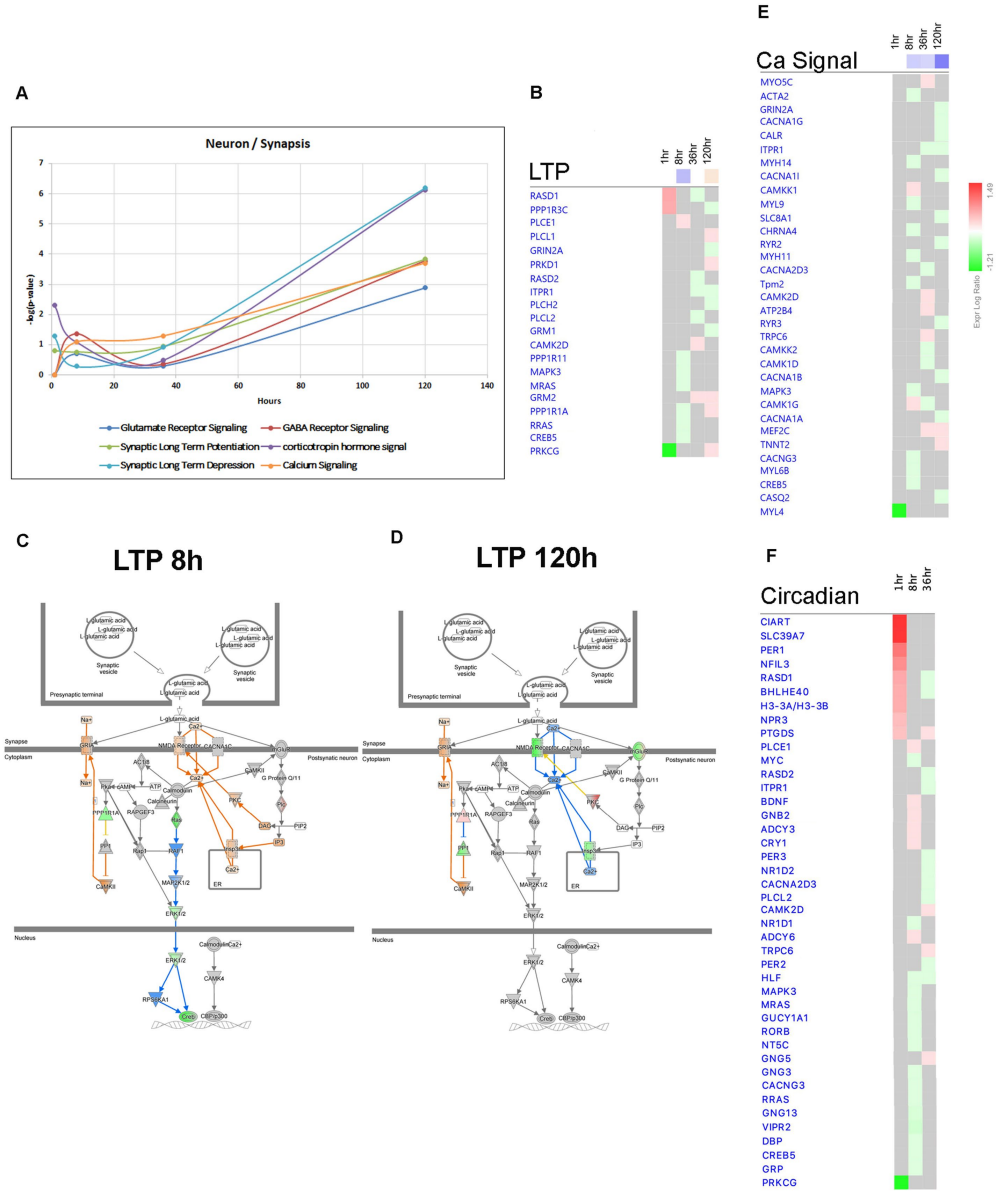
Temporal molecular changes in hippocampus after SE in pathways related to neuronal function and activity. **(A)** Temporal changes in Glutamate Receptor Signaling, Synaptic Long-Term Potentiation (LTP), Synaptic Long-Term Depression, GABA Receptor Signaling, Corticotropin Hormone Signal, and Calcium Signaling demonstrating late changes at 120-h time point. Y-axis on graph is-Log (*p*-value), where *p*-value is for significance of pathway at particular timepoint according to IPA analysis. **(B)** Temporal heatmap depicting the direction of change in gene expression for the Synaptic Long-Term Potentiation Pathway. **(C,D)** IPA Canonical Pathway, “LTP Pathway” at 8 h and 120 h after the onset of SE, respectively. **(E,F)** Temporal heatmap depicting the direction of change in gene expression for the Calcium Signaling Pathway and Circadian Signaling Pathway, respectively.

The conventional perspective on epilepsy has predominantly focused on the response of neurons to synchronized hyperactivity resulting from increased excitatory neurotransmission through glutamatergic signaling, and decreased inhibitory neurotransmission through GABAergic signaling (Ren and Curia, 2021). However, recent data suggest that other neuronal mechanisms may also be involved in the pathophysiology of epilepsy, such as suppression of calcium signaling (Conte et al., 2020) and alterations in synaptic scaffolding proteins (Needs et al., 2019). Consistent with this, our findings indicate that Canonical Pathways associated with Synaptic Long Term Depression (Figures 7A, 13A), Calcium Signaling (Figures 7A, 13A,E), and Synaptogenesis Signaling (Figures 7A, 13A) were largely downregulated after the 8-h time point, with the most pronounced effects observed at 120 h following pilocarpine injection (Figures 7A, 13A). Synaptic Long Term Potentiation was initially suppressed at 8 h, but subsequently activated at 120-h time point (Figures 7A–D). Additionally, dysregulation of GABA-Receptor Signaling was evident (Figure 13A). Interestingly, at the 120-h time point, the expression of genes encoding glutamatergic receptor subunits *Grin2a* and *Grm1* decreased, while the expression of genes encoding GABAergic receptor subunit *Gabre* increased, potentially in response to changing synaptic activity. Our findings corroborate the suppression of calcium signaling (Conte et al., 2020), as genes associated with this pathway, including *Adcy9, Cacna1a, Cacna1b, Cacna1g, Cacna1i, Kcnma1, Nos1, Pde1a,* and *Itpr1,* were predominantly downregulated at the 120-h time point (Figure 13E). Moreover, we observed downregulation of several genes associated with presynaptic vesicle cycling, such as *Syt12, Vamp1,* and *Syt4*, starting from 36 h after pilocarpine injection, while genes encoding postsynaptic density scaffolding proteins, including *Dlg2, Homer1, Shank1,* and *Shank3,* were downregulated at 120 h (Figure 13E).

Epilepsy in humans has been associated with disrupted circadian patterns (Baud et al., 2018) and recent studies in animal models of TLE have revealed dysregulation of circadian gene expression (Matos et al., 2018; Debski et al., 2020; Zhang et al., 2021). In our study, we observed temporal changes in circadian gene expression at 1, 8, and 36 h after pilocarpine injection. Our results demonstrate dysregulation of genes from Circadian Rhythm Signaling Pathway (Figures 2B, 7A), with upregulation of *Per1* (at 1 h), *Cry1* (at 8 h), and downregulation of *Per2* and *Per3* (at 36 h) (Figure 13F).

**Figure 14:**
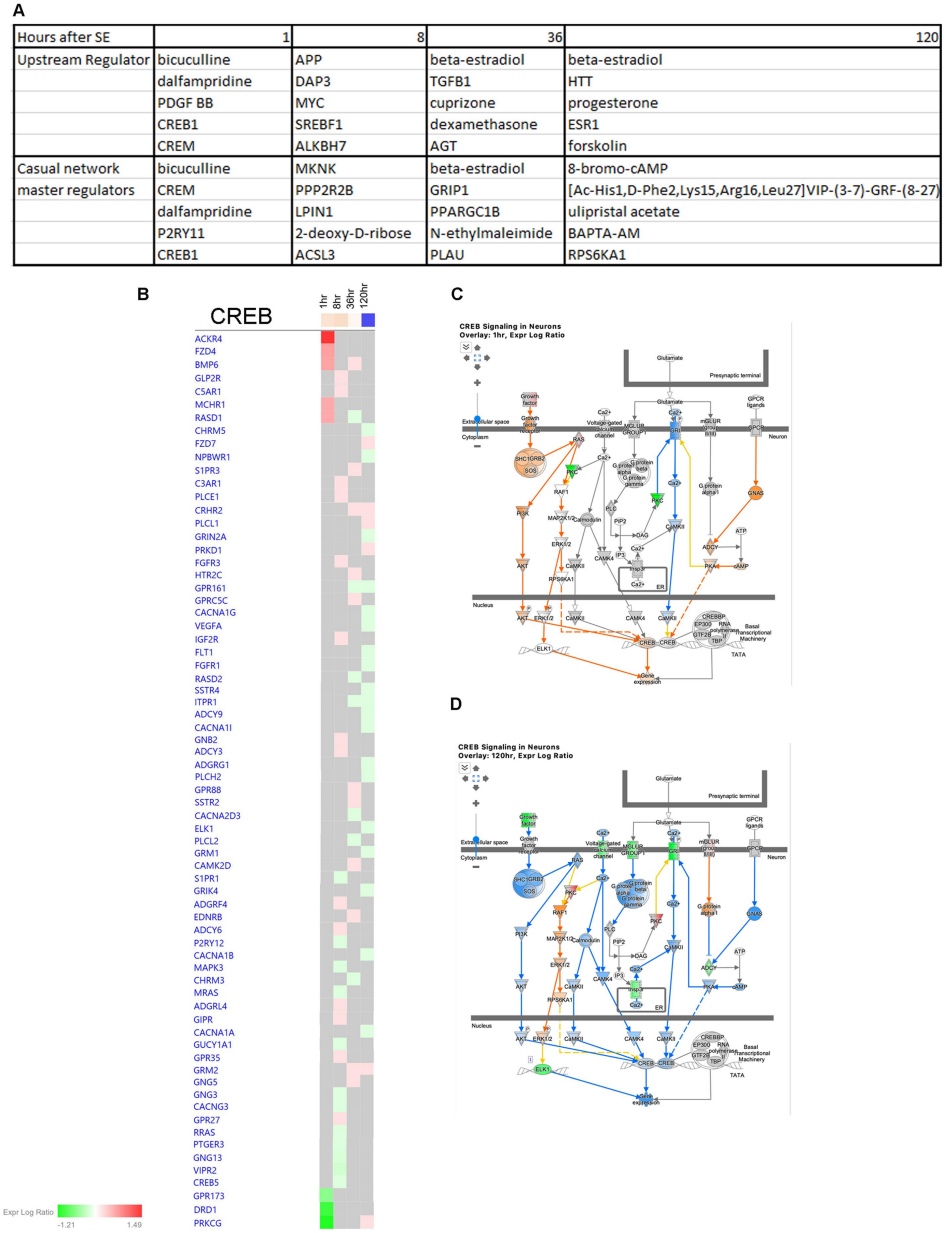
Predicted upstream regulators of hippocampus physiological changes after SE. **(A)** Predicted Upstream Regulator and Casual Network Master Regulator for each time point after SE in hippocampus. **(B)** Temporal heatmap depicting the direction of change in gene expression for the CREB Signaling Pathway. **(C,D)** Predicted regulation of CREB signaling canonical pathway cellular network at 1 h and 120 h after the onset of SE, respectively.

In order to identify potential therapeutic targets for intervention, we conducted an IPA Upstream Regulator Analysis and Causal Network Master Regulator Analysis (Figure 14A). These analyses helped us predict the upstream transcriptional regulators that may explain the observed changes in gene expression in our dataset, regardless of changes in their own gene expression levels. Our findings highlight CREB1 as a significant regulator at 1 h after the onset of seizure (Figure 14A). Notably, CREB signaling in neurons undergoes significant changes following SE, with upregulation observed at the 1- and 8-h time points (Figures 7A, 14B,C), and downregulation at 120 h (Figures 7A, 14B,D).

Several microRNAs exhibited changes in expression levels following seizures. A significant number of microRNAs were upregulated at both 1 h and 24 h after seizure, including *miR-1264-5p, 1,298-3p, 1,298-5p, 1912-3p, 204-5p, 448-3p, 34c-3p,* and *34c-5*. Some of these microRNAs have been previously associated with epilepsy or apoptosis in other studies (Cao et al., 2015; Ruibin et al., 2018; Organista-Juárez et al., 2019; Wang and Zhao, 2021). Another group or microRNAs showed upregulation at 1 h but downregulation at 8 h after seizure, including *miR-*6236 and 6,538. *MiR-5106,* which has been implicated in the healing process (Xiong et al., 2020), was downregulated at both 1 and 8 h. *MiR-7654-3p* was downregulated at both 8 and 24 h, while *miR-431-5*p exhibited upregulation at 24 h followed by downregulation at 120 h (Xie et al., 2022). *MiR-34b-5p*, which has been linked to inflammation, apoptosis, and Parkinson’s disease in previous studies (Liu et al., 2016; Xie et al., 2018; Li et al., 2020; Xie et al., 2022) showed changes in expression levels at all stages, with upregulation at early stages and downregulation at 120 h (Figure 5B), similar to another recent SE study (Li et al., 2022). It will be interesting to investigate specific biological significance of miR-34b-5p in this mouse model of TLE and this could be fruitful future direction for study.

The functions of many of these microRNAs remain unclear, and further work is needed to establish their functional relevance. To gain insights into the potential genes or pathways that may be regulated by these microRNAs, we utilized miRTargetLink 2.0 (Kern et al., 2021) on our data set. The results are presented in Supplementary Table S5. For example, *miR-1912-3p* was upregulated at 1 h, while one of its putative target genes, *Creb5,* was downregulated in our mRNA-seq analysis at 8 h. Similarly, m*iR-125a-5p* was downregulated at 24 h, while its known target gene, *Ptpn18,* was up-regulated in our mRNA-seq analysis at 36 h.

We further conducted an in-depth analysis to identify co-regulated groups of genes using the CEMitool package. Our finding revealed that module M2 exhibited a significant over-representation of genes associated with Epithelial-Mesenchymal Transition (EMT) pathways (Figures 7B,D). Further detailed analysis of the M2 module revealed that it predominantly contains genes whose expression is enriched in the tissues proximal to the hippocampus, the choroid plexus. This is most likely due to the inclusion of choroid plexus tissue during the isolation of the hippocampus, as previously demonstrated (Stankiewicz et al., 2015). However, we cannot completely rule out the possibility that the EMT pathway is indeed upregulated in hippocampus at the 1 h-time point and may potentially play a role in neuroprotection at later time points. Further studies, utilizing advanced spatial transcriptomics technologies (Williams et al., 2022) will be required to validate this hypothesis.

## Discussion

The administration of pilocarpine in mice causes various characteristic features of temporal lobe epilepsy (TLE), including a latent period and resistance to medication. Our primary objective is to elucidate the fundamental molecular mechanisms underlying epileptogenesis and identify key signaling pathways that contribute to recurrent seizures and subsequent neurodegeneration. Furthermore, we aim to identify pivotal pathways that play a neuroprotective role and could potentially serve as targets for developing new treatments for epilepsy and seizures. To achieve this, we conducted a comprehensive analysis at specific time points corresponding to different stages of the epileptic process, including the acute phase immediately after seizure onset (1 h), after status epilepticus (SE) (8 h), in the middle of the latent period (36 h), and at the end of the latent period just prior to the onset of spontaneous and recurrent seizures (120 h). In our study, we observed alterations in several pathways that are known to be implicated in epilepsy and seizure pathogenesis, such as IER genes, IGF1, ERK/MAPK, EIF2 signaling, oxidative stress, inflammation, changes in extracellular matrix, increased excitatory neurotransmission through glutamatergic signaling, and decreased inhibitory neurotransmission along with suppression of calcium signaling.

It is well-established that synchronized neuronal network firing during seizures results in an upregulation of IER genes, such as FOSB (Lösing et al., 2017), which acts as transcription factors and can subsequently trigger alterations in other molecular pathways in the hippocampus (Stephens et al., 2020; Galvis-Montes et al., 2023). However, limited data are available regarding the connections of other IER genes in this context. In our study, we identified a group of IER genes that were upregulated in the acute phase, and all of these genes belonged to module M4 (Figure 7E), which represents the TNF alpha signaling pathway. This pathway plays a critical role in immune and inflammatory responses, suggesting that IER transcription factors may be involved in the initiation of immune responses. For instance, *Gadd45b* and *Gadd45g* have been shown to be upregulated in hippocampus within 1 h after seizures (Henshall et al., 1999), and their activation in neurons has been implicated in functions such as proliferation, differentiation, adult neurogenesis, DNA-methylation, and apoptosis (Ma et al., 2009; Gavin et al., 2013). Our data demonstrate the upregulation of both *Gadd45b* and *Gadd45g* at the 1-h time point, with continued upregulation of *Gadd45g* at both 36- and 120-h time points. Recent studies have suggested a neuroprotective function for *Gadd45b* in ischemic neuronal death (Cho et al., 2019) and another study showed that *Gadd45g* acts through β-catenin to dynamically mediate a neurogenic/apoptotic fate switch during neuronal stem cells differentiation, where overexpression of *Gadd45g* promotes survival and neurogenesis, while loss of expression leads to apoptosis (Rosenbloom et al., 2020). Our results are consistent with the idea that the *Gadd45* IER gene may play a neuroprotective role in the context of seizures.

The IGF1 pathway has been known to enhance hippocampal excitatory and seizure activity through the activation of the ERK/MAPK pathways (Jiang et al., 2015). Our findings indicate that this pathway was already upregulated during the acute seizure phase (Figures 1C, 8A,B). The ERK/MAPK pathway has been implicated in SE, seizure, and epilepsy (Hansen et al., 2014; Gangarossa et al., 2015; Ahmed et al., 2021). Our results further reveal that alterations in both the IGF1 and ERK/MAPK pathways occur during the acute phase (Figures 1C,F,G, 8A,C,D). The ERK/MAPK pathway has multiple intracellular targets and is involved in the regulation of transcription and neuronal plasticity, potentially contributing to cell death, synaptic reorganization, and neuronal survival. For example, it has been demonstrated that ERK phosphorylation triggers the induction of expression of multiple IER genes during the acute phase in the kainic acid model of SE (Blüthgen et al., 2017).

The EIF2 pathway is critical for the integrated stress response, regulation of translation, and ribosome function (Bellato and Hajj, 2016; Costa-Mattioli and Walter, 2020). Previous studies have shown that certain genes from this pathway are regulated during SE and at later time points (Ghijsen et al., 2001; Carnevalli et al., 2006). Our data demonstrate that the EIF2 pathway was upregulated during the initial stages following pilocarpine injection, specifically at 1 and 8 h (Figures 2C, 10), and returned to normal levels at later time points.

The role of oxidative stress and mitochondrial dysfunction in the neuropathology of seizures, epilepsy, and SE is well established. Perturbation in the levels of reactive oxygen species (ROS) are a consequence of the overproduction of these radicals and decline in the function of antioxidant pathways (Olowe et al., 2020). The mitochondrial respiratory chain complexes are major sources of ROS production, and cytoplasmic oxygenase enzymes can also generate ROS. Antioxidant systems, including NRF2-mediated Oxidative Stress Response, Superoxide Radical Degradation, and Glutathione-mediated Detoxification, play a crucial role in mitigating oxidative stress (Kovac et al., 2017; Pearson-Smith and Patel, 2017). In our model of TLE, changes in genes responsible for major insults on mitochondrial function were observed at 8 h after pilocarpine injection (Figures 2C–E,G, 7A–C, 11). At this time point, multiple genes from complex I, III and IV of the mitochondrial respiratory chain were downregulated, resulting in an expected increase in oxidative stress. However, we observed that increases in antioxidant systems were much smaller compared to the mitochondrial genes (Figure 11), suggesting that oxidative stress might not be fully mitigated and could potentially trigger new seizures and continue to damage the hippocampus.

Inflammation is a hallmark of seizures (Marchi et al., 2014), but it remains unclear whether inflammation is a secondary response to neuronal damage or actively contributes to epileptogenesis. Furthermore, there is still ongoing debate regarding the role of microglia and infiltrating macrophages in seizures, with conflicting evidence on whether their activation is beneficial and neuroprotective, or harmful and exacerbates neurotoxicity (Tian et al., 2017). Some studies have suggested that microglia may be less proinflammatory than myeloid infiltrates during SE (Vinet et al., 2016). Immune responses to seizures are typically delayed by 24–48 h from the onset of SE (Eyo et al., 2017), and it has been proposed that activation of the JAK/STAT pathway leads to induction of the inflammatory response (Okamoto et al., 2010; Tian et al., 2017). Our data revealed that the JAK/STAT pathway and TNFA signaling pathway via NF-kB were upregulated as early as 1 h after pilocarpine injection and remained elevated at 8 h (Figures 7B,C, 12C). Subsequently, at 36 h, pathways associated with Acute Phase Response, Complement System, and P2Y-purinergic Receptor Pathways, all involved in inflammation and immune responses, showed increased expression and remained elevated (Figure 12C). Interestingly, our findings showed that proinflammatory markers were predominantly upregulated in the early stages after SE, while protective and pro-survival genes were upregulated at 36 h (Figure 12D), suggesting a temporal shift in the gene expression pattern during epileptogenesis.

Seizures and epilepsy can compromise BBB function and induce leakage (Fabene et al., 2008; Han et al., 2019; Lochhead et al., 2020; Löscher and Friedman, 2020). Previous studies using the rat pilocarpine model of TLE have reported downregulation of tight junction proteins and upregulation of matrix metalloproteinases *Mmp2 and Mmp9* in brain capillary tissue after 48 h, leading to BBB dysfunction in epilepsy (Rempe et al., 2018). However, in contrast to these findings, gene expression analysis of rat and mouse hippocampal samples from pilocarpine and kainic acid epilepsy models indicated upregulation of multiple genes involved in ECM/integrin signaling and tight/gap junctions after 24 h (Dingledine et al., 2017; Han et al., 2019). Our data demonstrated that changes in ECM genes and BBB-related genes mostly occurred at the 36-h time point (Figures 3C,D, 12B), with upregulation of collagen genes from the GP6 signaling pathway, claudins (genes for tight junction complex), ECM/Integrin signaling, and gap junction genes, suggesting a potentially neuroprotective nature of increased expression of these genes. In contrast, we observed downregulation of important collagen genes at the 120-h time point, which coincides with the end of latent phase and could potentially contribute to seizure occurrence. These findings are consistent with studies reporting that mutations in genes encoding these proteins in humans can result in functional loss and drive neurological phenotypes including epilepsy (Zagaglia et al., 2018).

The most widely accepted understanding of the underlying mechanism of epilepsy centers around aberrant neuronal activity resulting from its synchronized hyper activity due to increased excitatory neurotransmission via glutamatergic signaling, and decreased inhibitory neurotransmission via GABAergic signaling (Ren and Curia, 2021). Recent findings suggest other neuronal mechanisms may also play a role in the pathophysiology of epilepsy, such as suppression of calcium signaling (Conte et al., 2020) and alterations in synaptic scaffolding proteins (Needs et al., 2019). Our own data indicate that changes in gene expression related to glutamate receptor signaling, GABA-receptor signaling, calcium signaling, synaptic long-term potentiation and depression, predominantly occur during the later phase of the latent period, specifically at the 120-h time point following pilocarpine injection (Figure 13). In general, our findings suggest a shift in gene expression from excitatory to inhibitory synapses during this latent period, with downregulation of genes associated with glutamatergic synapses and upregulation of genes associated with GABAergic synapses. Additionally, downregulation of genes related to post-and pre-synaptic proteins and calcium signaling is a characteristic feature of synaptic inhibition towards the end of the latent period.

In addition to these well-established metabolic pathways implicated in the response to SE, we have identified several novel or less-known pathways that may be involved, including dysregulation of circadian signaling pathway (Figure 13C), alterations in iron homeostasis (Figures 9A,B), changes in BMP signaling pathway (Figures 9C,D), ferroptosis (Figure 9A), and upregulation of cholesterol biosynthesis (Figures 12A,B).

We have observed dysregulation of the iron homeostasis signaling pathway at 1- and 8-h after pilocarpine injection (Figures 1C, 9B). The role of iron metabolism in epilepsy is still being elucidated (Cai and Yang, 2021), with evidence of iron accumulation in the hippocampus of patients with TLE and SE (Zimmer et al., 2020, 2021). Ferroptosis, a newly discovered pathway that involves the accumulation of iron inside cell leading to lipid peroxidation and increased ROS production, has been detected during epilepsy in previous studies (Kahn-Kirby et al., 2019; Mao et al., 2019b). Recent research has also shown that iron homeostasis could be regulated by bone morphogenic proteins (BMP) in non-neuronal tissue (Muckenthaler et al., 2017; Xiao et al., 2020), and BMP proteins in general have been implicated in neuroprotection through participation in neurogenesis. The BMP/SMAD signaling pathway, which is involved in hepcidin regulation, a key regulator protein for iron entry into circulation (Ganz, 2003), showed changes in gene expression in our study (Figures 9C,D), suggesting a potential role for ion regulation/BMP pathways in epileptogenesis and as a novel target for seizure treatment.

Cholesterol, an essential structural component of myelin and cellular membranes in the brain, is synthesized autonomously in the brain due to the BBB preventing its influx from the blood (Orth and Bellosta, 2012; Zhang and Liu, 2015). Cholesterol production and regulation is cell-type specific, with astrocytes being recognized as major producers of lipids that are transported to neurons and play a role in regulating synapse development and function (van Deijk et al., 2017). Cholesterol biosynthesis involves more than 20 enzymes participating in over 30 enzymatic reactions (Waterham, 2006; Mitsche et al., 2015). However, data on sterol levels in neurodegeneration and excitotoxicity models are limited and conflicting (Kim et al., 2010; Ong et al., 2010; Heverin et al., 2012; Chali et al., 2019). Recent work on human hippocampus and cerebrospinal fluids (CSF) from SE patients demonstrated alterations in cholesterol metabolism, with increased levels of cholesterol and desmosterol, but decreased levels of 24-hydroxycholesterol, the hydroxylated derivative of cholesterol (Hanin et al., 2021). In the present study, we found upregulation of 10 genes involved in cholesterol biosynthesis at 36 h after the onset of seizures (Figure 12A), suggesting increased cholesterol production. We also observed upregulation of genes involved cholesterol transport within the brain at 36 h, with some genes remaining elevated at 120 h. We propose that changes in cholesterol metabolism are a significant late component of the hippocampal response to seizures. Notably, upregulation of *Dhcr24*, the gene encodes 24-Dehydrocholesterol reductase, a key synthetase in cholesterol synthesis, has been shown to reduce loss of neurons under inflammatory conditions (Martiskainen et al., 2017), suggesting a potential neuroprotective role of increased cholesterol production.

**Figure 15:**
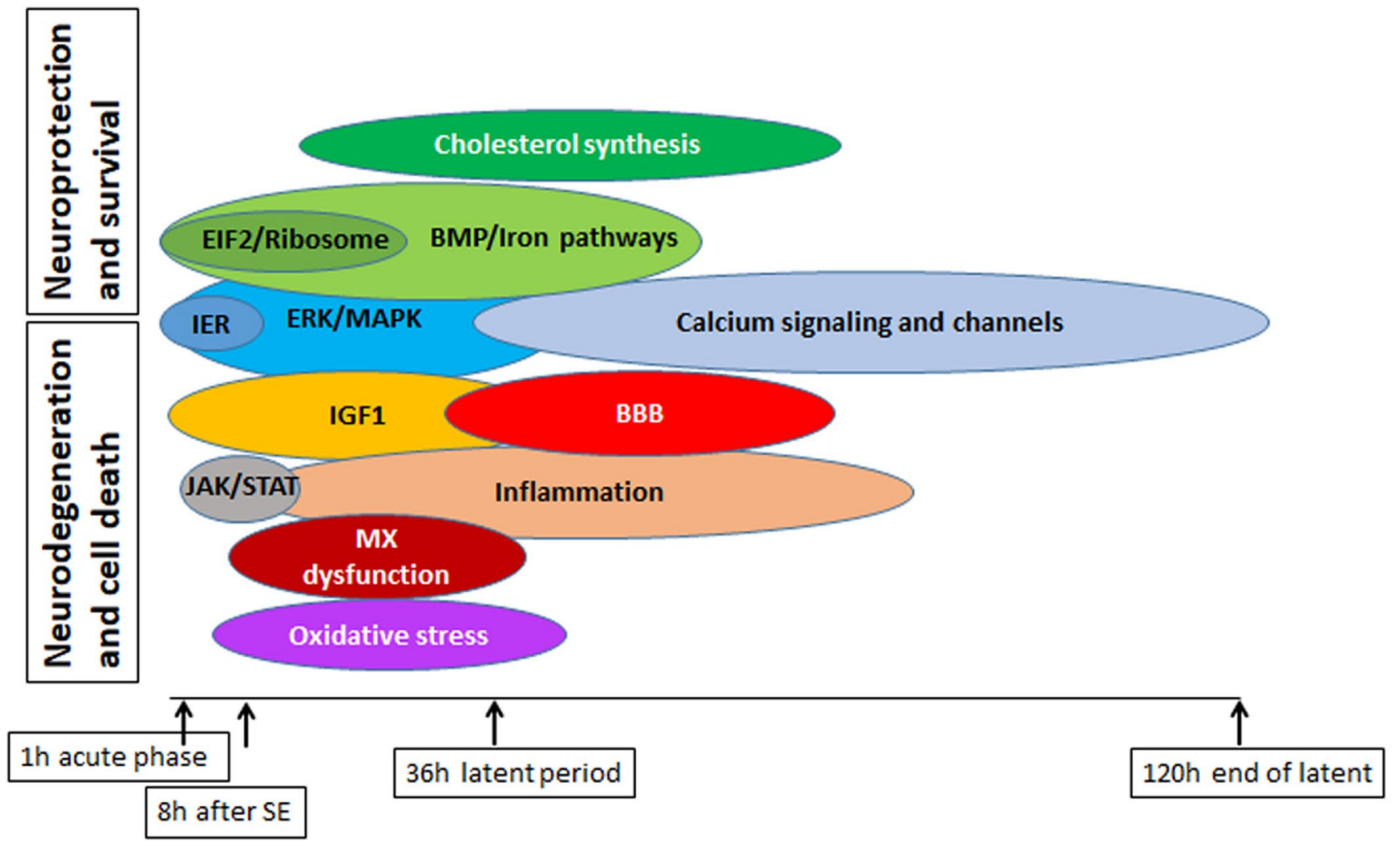
Summary of major pathways involved in physiological response to SE in mouse hippocampus, with emphasis on their potential deleterious or beneficial effects on cell survival and protection.

Epilepsy is a prevalent neurological disorder; however, despite extensive research efforts, approximately 30% of patient cases remain refractory to treatment. Our study of different physiological pathways implicated in epileptogenesis (Figure 15) has yielded a deeper understanding of the underlying molecular mechanisms and signaling pathways that contribute to neurodegeneration and subsequent seizure development, as well as the neuroprotective pathways and their interplay. Our analysis of Upstream Regulators and Causal Network Master Regulators by IPA has identified several potential avenues for new therapeutic interventions. Remarkably, the CREB1 pathway appears to be particularly promising, and targeting alteration of the CREB pathway may hold potential as a new therapeutic approach.

## Data availability statement

The data presented in the study have been deposited in the NCBI GEO repository with the accession number GSE198498, and have been released.

## Ethics statement

The animal study was approved by Pennsylvania State University College of Medicine Institutional Animal Care and Use Committee (protocol #46432). The study was conducted in accordance with the local legislation and institutional requirements.

## Author contributions

EP: Writing – review & editing, Writing – original draft, Validation, Investigation, Formal analysis, Conceptualization. YK: Writing – review & editing, Visualization, Software, Methodology, Formal analysis, Data curation. ML: Writing – review & editing, Software, Formal analysis, Data curation. CB: Writing – review & editing, Writing – original draft, Supervision, Project administration, Methodology, Funding acquisition, Conceptualization.

## Footnotes

^1^http://hannonlab.cshl.edu/fastx_toolkit

## Supplementary material

SUPPLEMENTARY FIGURE 1 Additional to Figures 1–4. Volcano plots showing differentially expressed genes at 1hour **(A)**, 8 hours **(B)**, 36 hours **(C)** and 120 hours **(D)** after SE. Only significant genes (at *p*-value < 0.05) are shown in the plots.

SUPPLEMENTARY FIGURE 2 Additional to Figure 5. Western blots for selected protein. β-actin was used as the loading control.
