# Supplementary figures and images for: Temporal changes in mouse hippocampus transcriptome after pilocarpine-induced seizures
Source: Front Neurosci. 2024 Jul 8;18:1384805. doi: 10.3389/fnins.2024.1384805 (PMC11260795; doi:10.3389/fnins.2024.1384805)

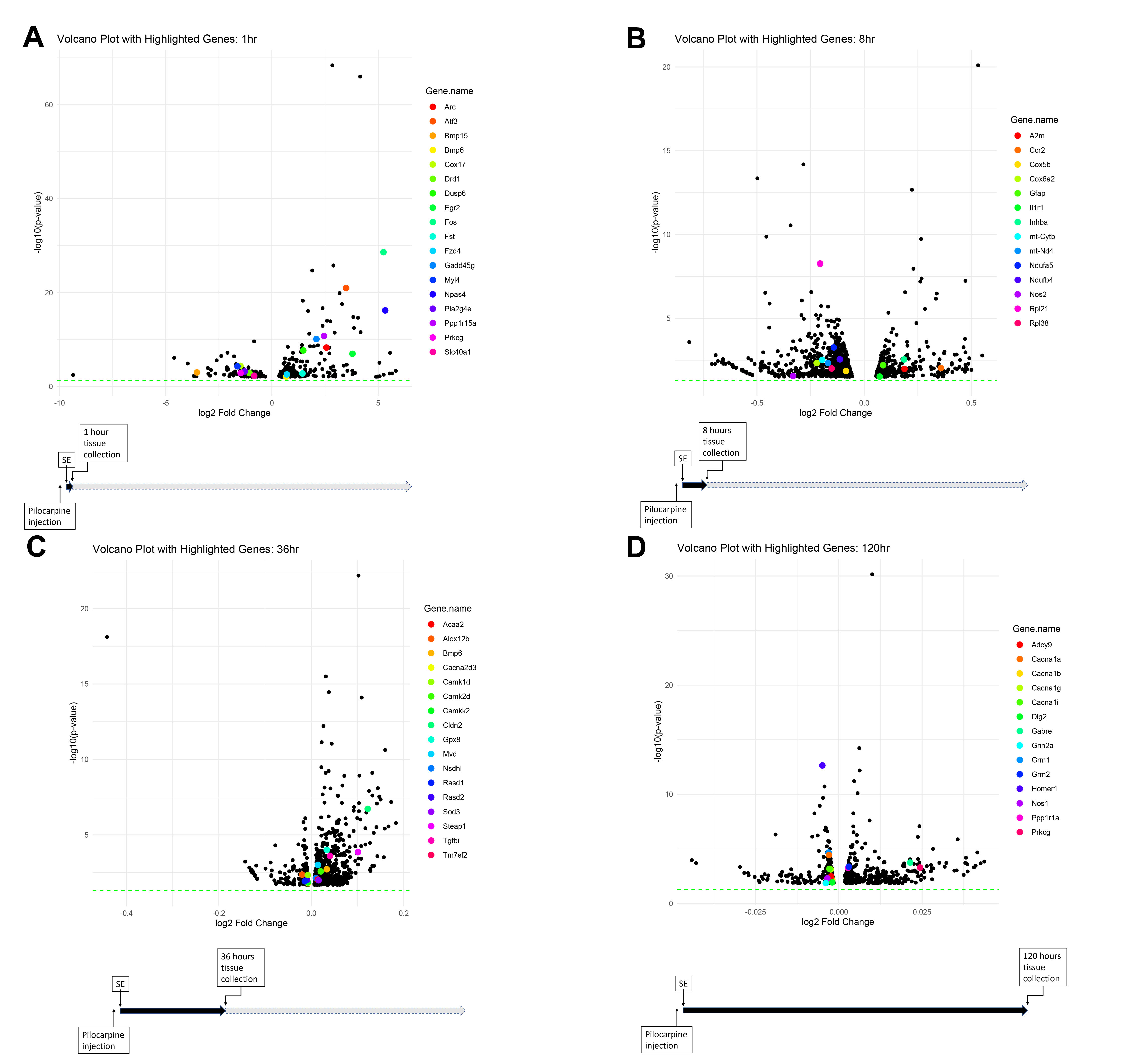

Supplement: SUPPLEMENTARY FIGURE 1 — Additional to Figures 1–4. Volcano plots showing differentially expressed genes at 1hour (A), 8 hours (B), 36 hours (C) and 120 hours (D) after SE. Only significant genes (at p-value < 0.05) are shown in the plots. [file Image_1.JPEG]

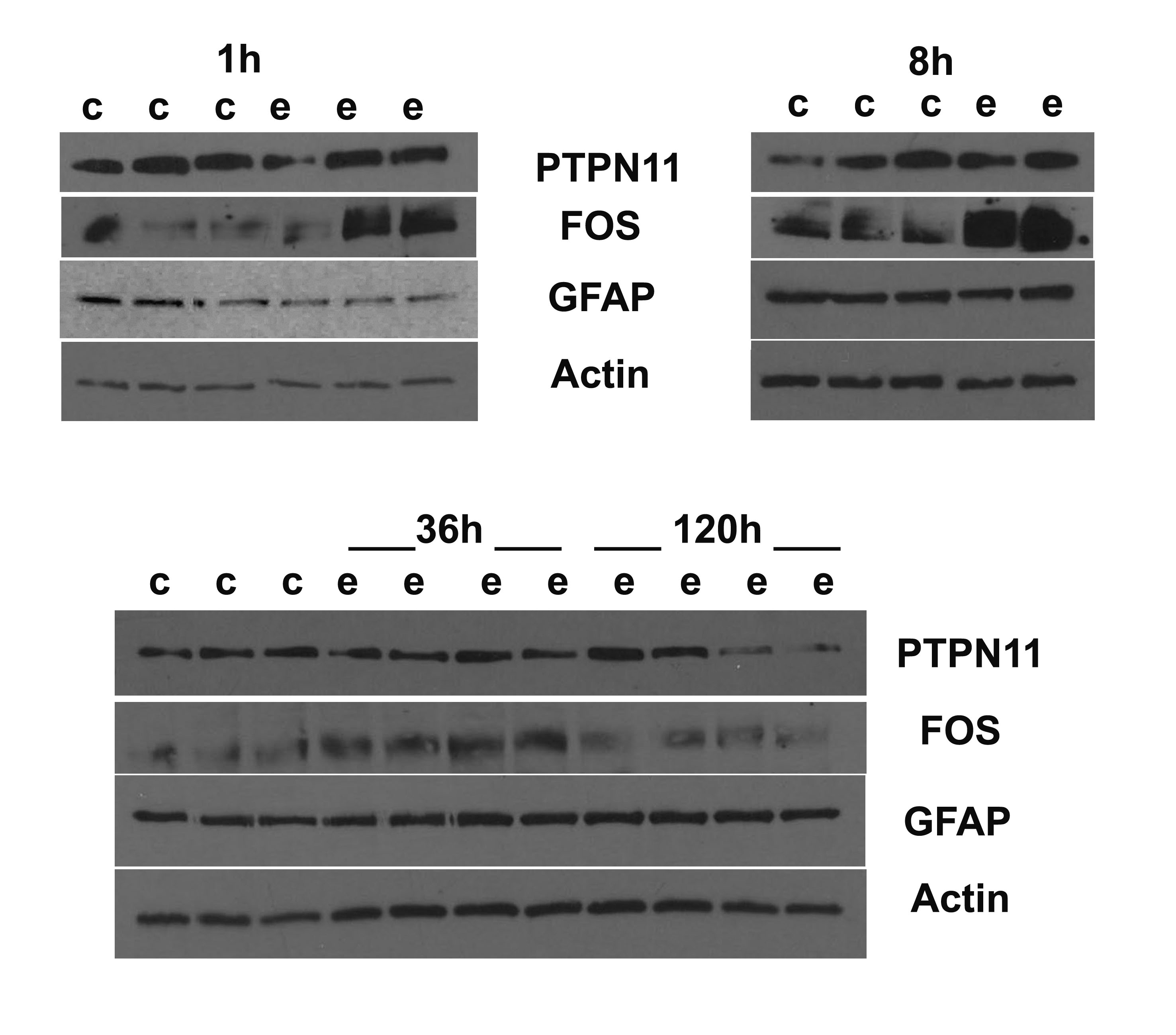

Supplement: SUPPLEMENTARY FIGURE 2 — Additional to Figure 5. Western blots for selected protein. β-actin was used as the loading control. [file Image_2.JPEG]
