# Supplementary material for: Temporal changes in mouse hippocampus transcriptome after pilocarpine-induced seizures
Source: Front Neurosci. 2024 Jul 8;18:1384805. doi: 10.3389/fnins.2024.1384805 (PMC11260795; doi:10.3389/fnins.2024.1384805)
Supplement: Supplementary file 6 [file Table_4.DOCX]

| **Table 2. Number of small RNA that have change in expression level** | | | | | | |
| --- | --- | --- | --- | --- | --- | --- |
| **between control and seizure animals with p<0.05.** | | | | |  |  |
| **number smRNA** | **overall** | **piRNA** | **microRNA** | **Snor-RNA** | **novel** | **ribosome** |
| **1 hour** | 52 | 27 | 19 | 4 | 2 | 0 |
| **8 hours** | 64 | 38 | 12 | 10 | 1 | 3 |
| **24 hours** | 54 | 13 | 32 | 5 | 3 | 1 |
| **5 days** | 38 | 7 | 10 | 19 | 1 | 1 |
