# Supplementary material for: Temporal changes in mouse hippocampus transcriptome after pilocarpine-induced seizures
Source: Front Neurosci. 2024 Jul 8;18:1384805. doi: 10.3389/fnins.2024.1384805 (PMC11260795; doi:10.3389/fnins.2024.1384805)
Supplement: Supplementary file 5 [file Table_2.DOCX]

qRT-PCR Primers.		
	**FORWARD**	**REVERSE**
Inhba	AGGACGACATTGGCAGGAGG	CTGGTTCTGTTAGCCTTGGGG
Otx2	TCGCCACCTCTACTTTGATAG	AGCCGCATTGGACGTTAG
Gfap	GAGAGAAAGGTTGAATCGCTGG	CGGCGATAGTCGTTAGCTTC
Gapdh	Qiagen QT01658692	
