# Supplementary material for: Temporal changes in mouse hippocampus transcriptome after pilocarpine-induced seizures
Source: Front Neurosci. 2024 Jul 8;18:1384805. doi: 10.3389/fnins.2024.1384805 (PMC11260795; doi:10.3389/fnins.2024.1384805)
Supplement: Supplementary file 6 [file Table_5.DOCX]

	**UP**		
	**DOWN**		
	microRNA	Function	Presumable gene targets
	**1h**		
	**mmu-miR-1195**		2810006K23Rik(8),Arvcf (8,36), Bach2(36), Brat1(8), Depdc5(120), Fzd7(120), Galntl6(36,120), Gtpbp3(8), Iqcg,(1) Kl(1,36,120), Nploc4(8), Ooep(8,36), Plekhh(36), Polr3gl(8), Rpl31(8),Scd4(8), Sema3e(36,120), Senp5(8), Sgk3(8), Slc28a3(120), Smpd4(120), Tapbp(36),Usp45,(120) Zfp113(1)
a	**mmu-miR-1264-5p**	no	no
b	**mmu-miR-1298-3p**	epilepsy patient	no
c	**mmu-miR-1298-5p**	regulation of cardiachypertrophy, epilepsy patient	Loxl2(36), Slc16a9(36)
	**mmu-miR-143-3p**	MAPK IL-1, insulin, WNT - signaling, BBB	ELK1(120),Myo5c(36),Ogfrl1(8), Ptn(8), Zfp488(8)
d	**mmu-miR-1912-3p**	calcium channel, pro-apoptotic	C3ar1(8), Creb5(8), Ezr(36), Slc25a42(8)
e	**mmu-miR-204-5p**	pro-apoptotic	Dcx(36,120), Irs4(36), Itpripl2(36), Mrpl41(8), Rasd2(36), Rs1(36), Sox11(36)
	**mmu-miR-27a-5p**	AKT/IL-1 , membrane, mTOR, neuronal development, brain trauma	Prdm16(36)
f	**mmu-miR-34b-5p**	pro-apoptotic	Bcl6(36), Akap6(36,120), Angptl2(1,36,120), Arl15(120), Arrdc3(1), Bdnf(8), Cbfb(8), Dcx(36,120), Ddi2(8), Dock9(36,120), Epha8(120), Fam101b(36), Fam57a(8), Foxp1(120), Gpr173(1), Gpr56(120), Hpcal1(120), Iqgap2(36), Kcnq2(120), Med22(8), Mfsd2a(1), Neurod1(8), Pcdh891),Prkd1(8,36), Ptpru(36,120), Rab15(120), Rab8b(120), Rorb(8), Sdc4(8), Sgk3(8), Shisa6(36), Stac2((120), Syt4(120), Tbc1d2(36),Trpm3(36), Zfp174(1), Zfp532(120), Zfp579(36)
g	**mmu-miR-34c-3p**	epilepsy patient	no
h	**mmu-miR-34c-5p**	polII,regeneration, epilepsy patient	Nanos2(120), Bcl6(36), Med22(8), Notch1(8), Sgk3(8), Tbc1d2(36)
	**mmu-miR-375-3p**	epilepsy patient	Yap1(36), Elavl4((8), Adipor2(8), Rpsa(8)
i	**mmu-miR-448-3p**	lysosomal transport	Akap7(120), Ccdc82(8), Gjc3(8,36), Igf2(120), Sel1l(120), Tll1(8), Zfp488(8)
j	**mmu-miR-5106**	Purine metabolism	Lars2(8,36), Senp5(8)
	**mmu-miR-5126**		Bach2(36)
k	**mmu-miR-6236**	no	no
	**mmu-miR-6240**	no	no
l	**mmu-miR-6538**	no	no
	**mmu-miR-764-3p**	polyamine metabolic process	Slc25a39(36)
	**8h**		
	**mmu-miR-122-5p**	external side of plasma membrane, CAM, ischemia	Tmem50b(120), Sbk1(120), Hist1h1c(36) Myc(8), Bach1((8), Hfe(36), Tfrc(8), Zfp113(1)
f	**mmu-miR-34b-5p**	pro-apoptotic	Bcl6(36), Akap6(36,120), Angptl2(1,36,120), Arl15(120), Arrdc3(1), Bdnf(8), Cbfb(8), Dcx(36,120), Ddi2(8), Dock9(36,120), Epha8(120), Fam101b(36), Fam57a(8), Foxp1(120), Gpr173(1), Gpr56(120), Hpcal1(120), Iqgap2(36), Kcnq2(120), Med22(8), Mfsd2a(1), Neurod1(8), Pcdh891),Prkd1(8,36), Ptpru(36,120), Rab15(120), Rab8b(120), Rorb(8), Sdc4(8), Sgk3(8), Shisa6(36), Stac2((120), Syt4(120), Tbc1d2(36),Trpm3(36), Zfp174(1), Zfp532(120), Zfp579(36)
j	**mmu-miR-5106**	Purine metabolism	Lars2(8,36), Senp5(8)
	**mmu-miR-5112**	Serotonin and anxiety	Csmd3(36), Arid5a(1)
k	**mmu-miR-6236**	no	
	**mmu-miR-6369**	no	
l	**mmu-miR-6538**	no	
	**mmu-miR-6938-3p**	no	
	**mmu-miR-7057-5p**	no	
m	**mmu-miR-7654-3p**	no	
	**hsa-miR-6087**	no	
	**hsa-miR-7641**	no	
	**24 h**		
	**mmu-let-7b-5p**	brain injuries, seizure, anti-apoptotic	Bsg(36), Slc9a2(36), Slc24a2(36),Slc23a2(8), Itga4(8), Crk(8), Cd164(1)
	**mmu-let-7c-5p**	brain injuries, seizure, anti-apoptotic	Myc(8), Meis2(8)
	**mmu-miR-10b-5p**		Klf4(1),Cdkn1a(1,8), Rhbdl3(36), Hapln4(120), Kif3a(8), Nsl1(8)
	**mmu-miR-125a-5p**	Cytokines and Inflammatory response, pro-apoptotic	Stat3(8), Ptpn18(36), Lars2(8,36)
	**mmu-miR-1264-3p**		Steap3(36), Zfp113(1), Stk32a(1), Plce1(8), Slc7a11(36), Creb5(8), Ankrd52(120), Klhl2498)
a	**mmu-miR-1264-5p**	no	no
b	**mmu-miR-1298-3p**	epilepsy patient	no
c	**mmu-miR-1298-5p**	regulation of cardiachypertrophy, epilepsy patient	Loxl2(36), Slc16a9(36)
	**mmu-miR-137-3p**		Slc16a9(36)
	**mmu-miR-149-5p**	IL-1 signaling	Ipcef1(36), PRDM16(36), B3galt5(36), Vldlr(120), Sash1(120), Dpy19l3(120), 4933426M11Rik(36), Dgcr2(8), Txnip(1,120)
	**mmu-miR-182-5p**	ubiquitin,metal, cytokine pathways	Slc14a2(36), Srrm1(8)
	**mmu-miR-183-5p**	WNTsignaling, pluropotency pthway	no
	**mmu-miR-190a-5p**		Tcp11l1(8), Arl15(80, 9430020K01Rik(120), Cav2(36) Clk1(1), L1cam(8)
d	**mmu-miR-1912-3p**	calcium channel, pro-apoptotic	C3ar1(8), Creb5(8), Ezr(36), Slc25a42(8)
e	**mmu-miR-204-5p**	pro-apoptotic	Dcx(36,120), Irs4(36), Itpripl2(36), Mrpl41(8), Rasd2(36), Rs1(36), Sox11(36)
	**mmu-miR-219a-2-3p**	no	no
	**mmu-miR-221-3p**		Arvcf(8,36), Ddit498,120), Rs1(36), Timp3(120)
	**mmu-miR-320-3p**		Bicd2(120), Fosl2(1), Hspb6(36), Spry4(36)
	**mmu-miR-335-3p**		Ccdc153(8), Foxp1(120), Il17re,(36) Mat2a(36,120), Meis2(8), Rsad2,(8) Sel1l(120), Sfrp1(36,120), Sox11(36), Tes(36), Tmem38b(8)
	**mmu-miR-335-5p**	ER, Plasma membrane, antigen present	no
	**mmu-miR-34b-3p**	cilium,cell projection membr, cell-cell	Fbxo45(120), Tsc22d3(120), Ttc19(36)
f	**mmu-miR-34b-5p**	pro-apoptotic	Bcl6(36), Akap6(36,120), Angptl2(1,36,120), Arl15(120), Arrdc3(1), Bdnf(8), Cbfb(8), Dcx(36,120), Ddi2(8), Dock9(36,120), Epha8(120), Fam101b(36), Fam57a(8), Foxp1(120), Gpr173(1), Gpr56(120), Hpcal1(120), Iqgap2(36), Kcnq2(120), Med22(8), Mfsd2a(1), Neurod1(8), Pcdh891),Prkd1(8,36), Ptpru(36,120), Rab15(120), Rab8b(120), Rorb(8), Sdc4(8), Sgk3(8), Shisa6(36), Stac2((120), Syt4(120), Tbc1d2(36),Trpm3(36), Zfp174(1), Zfp532(120), Zfp579(36)
g	**mmu-miR-34c-3p**	epilepsy patient	no
h	**mmu-miR-34c-5p**	polII,regeneration, epilepsy patient	Nanos2(120), Bcl6(36), Med22(8), Notch1(8), Sgk3(8), Tbc1d2(36)
	**mmu-miR-3963**	Hedgehog Signaling Pathway	no
n	**mmu-miR-431-5p**	no	C3ar1(8), Elfn1(8), Senp5(8), Sgms2(1)
i	**mmu-miR-448-3p**	lysosomal transport	Akap7(120), Ccdc82(8), Gjc3(8,36), Igf2(120), Sel1l(120), Tll1(8), Zfp488(8)
	**mmu-miR-448-5p**	no	Lamp2(36)
m	**mmu-miR-7654-3p**	no	no
	**mmu-miR-92a-3p**	no	Btg2(1,8), Gm5148(8), Rhbdl3(36), Shox2(8)
	**mmu-miR-96-5p**	Serotonin and anxiety, estrogen	Gstm1(36), Slc14a2(36), Srrm1(8)
	**mmu-miR-99a-5p**	WNTsignaling, pluropotency pthway	no
	**5 days**		
	**mmu-miR-154-5p**	WNTsignaling, pluropotency pthway	no
	**mmu-miR-345-5p**	One Carbon Metabolism	Lsm12(8)
f	**mmu-miR-34b-5p**	pro-apoptotic	Bcl6(36), Akap6(36,120), Angptl2(1,36,120), Arl15(120), Arrdc3(1), Bdnf(8), Cbfb(8), Dcx(36,120), Ddi2(8), Dock9(36,120), Epha8(120), Fam101b(36), Fam57a(8), Foxp1(120), Gpr173(1), Gpr56(120), Hpcal1(120), Iqgap2(36), Kcnq2(120), Med22(8), Mfsd2a(1), Neurod1(8), Pcdh891),Prkd1(8,36), Ptpru(36,120), Rab15(120), Rab8b(120), Rorb(8), Sdc4(8), Sgk3(8), Shisa6(36), Stac2((120), Syt4(120), Tbc1d2(36),Trpm3(36), Zfp174(1), Zfp532(120), Zfp579(36)
	**mmu-miR-3547-3p**	no	no
n	**mmu-miR-431-5p**	no	C3ar1(8), Elfn1(8), Senp5(8), Sgms2(1)
	**mmu-miR-451a**	no	Mif(8,36) Myc(8), Thbd(36,120)
	**mmu-miR-503-5p**	pol II , Oxidative Damage, negative regulation of apoptotic signaling pathway	Creb5(8),Taok3(8)
	**mmu-miR-544-5p**	no	Cpm(36), Slc24a2(36)
	**mmu-miR-6481**	no	no
	**hsa-miR-4443**	mo	Espl1(36),Fam129b(8,36),Fblim1(36), Fkbp5(8), Polg(8), Rpl28(8), Trim72(8), Txnip(1,120)
